# Stem Cell-Based Therapy for Experimental Ischemic Stroke: A Preclinical Systematic Review

**DOI:** 10.3389/fncel.2021.628908

**Published:** 2021-04-14

**Authors:** Xi-Le Zhang, Xiao-Guang Zhang, Yan-Ran Huang, Yan-Yan Zheng, Peng-Jie Ying, Xiao-Jie Zhang, Xiao Lu, Yi-Jing Wang, Guo-Qing Zheng

**Affiliations:** Department of Neurology, The Second Affiliated Hospital and Yuying Children’s Hospital of Wenzhou Medical University, Wenzhou, China

**Keywords:** stem cell-based therapy, ischemic stroke, middle cerebral artery occlusion, neuroprotection, pre-clinical systematic review

## Abstract

Stem cell transplantation offers promise in the treatment of ischemic stroke. Here we utilized systematic review, meta-analysis, and meta-regression to study the biological effect of stem cell treatments in animal models of ischemic stroke. A total of 98 eligible publications were included by searching PubMed, EMBASE, and Web of Science from inception to August 1, 2020. There are about 141 comparisons, involving 5,200 animals, that examined the effect of stem cell transplantation on neurological function and infarct volume as primary outcome measures in animal models for stroke. Stem cell-based therapy can improve both neurological function (effect size, −3.37; 95% confidence interval, −3.83 to −2.90) and infarct volume (effect size, −11.37; 95% confidence interval, −12.89 to −9.85) compared with controls. These results suggest that stem cell therapy could improve neurological function deficits and infarct volume, exerting potential neuroprotective effect for experimental ischemic stroke, but further clinical studies are still needed.

## Introduction

Stroke is one of the leading causes of death and the first cause of acquired morbidity and mortality worldwide (Strong et al., 2007). Ischemic stroke is the most common type of stroke, defined as a syndrome characterized by a rapid onset of central nervous system function damage due to an interruption of the cerebral blood flow (Baker et al., 1998). However, recombinant tissue plasminogen activator (rtPA) remains the only pharmacological treatment approved by the Food and Drug Administration for thrombolysis in patients suffering from ischemic stroke (Wright et al., 2012; Jauch et al., 2013). Unfortunately, the relatively short therapeutic window of 3-4.5 h of rtPA and the risk of the devastating symptomatic intracranial hemorrhage limit its application (Chapman et al., 2014). Although the treatment protocols for acute ischemic stroke have been fundamentally altered because of the updated guidelines using endovascular techniques in Powers et al. (2015), this guideline concluded that certain endovascular procedures have been demonstrated to provide clinical benefit in selected acute ischemic stroke patients within a slightly prolonged therapeutic time window of 6 h after symptom onset. Thus, given the gap between the widespread occurrence of the disease and the limitations of conventional therapies available, novel intervention for ischemic stroke is urgently needed.

An interest in stem cell-based therapy was spawned by the limited eligibility for thrombolysis and failure of the neuroprotective paradigm (Jakala and Jolkkonen, 2012). Stem cells are undifferentiated biological cells that have the capacity to proliferate and differentiate into mature specialized cells and can divide to produce more stem cells (Mora-Lee et al., 2012). Various types of stem cells, including neural stem/progenitor cells (NSCs), embryonic stem cells, immortalized pluripotent stem cells (iPSCs), and tissue-derived stem cells such as mesenchymal stem cells (MSCs) and bone marrow mononuclear cells, have attained tremendous attention as a promising approach in nervous system diseases as disparate as motor neuron disease (Cabanes et al., 2007; Su et al., 2009), Parkinson’s disease (Yasuhara et al., 2006), multiple sclerosis (Aharonowiz et al., 2008; Morando et al., 2012), and stroke (Jensen et al., 2013; Wang J. et al., 2013). There is now considerable preclinical literature on the possible benefits of stem cell transplantation following ischemic stroke. Stem cell may assist stroke recovery through cell replacement, neuroprotection, angiogenesis, endogenous neurogenesis, and modulation on inflammation and immune response (Hao et al., 2014).

To better foster the stem cell-based therapies that progressed into clinical trials, we need to further understand the optimal requirements for stem cell administration to improve the therapeutic effects on ischemic stroke. However, the ideal type of stem cell and from what donor species and tissue source, the appropriate time for injections, the number of cells needed, and the best administration route are still not clear. Furthermore, whether reports of efficacy in animal models are potentially biased in favor of positive results and whether the magnitude of integrative and protective effects is large enough to be potentially clinically meaningful are worth further investigation. Systematic review of preclinical data is a powerful analytical tool typically used to offer the most objective evidence for the efficacy of a treatment and improve the likelihood of success of future clinical trials (Murphy and Murphy, 2010). This systematic review included controlled studies of stem cell therapy in animals exposed to stroke compared with placebo control or no treatment in stroke animals, in which the outcome was measured with neurological function score and infarct size/infarct volume. The purpose is to evaluate the safety and efficacy of stem cell therapy for ischemic stroke and confirm the conditions of greatest efficacy.

## Methods

We strictly obeyed the Preferred Reporting Items for Systematic Reviews and Meta-Analyses: The PRISMA Statement (Moher et al., 2010) to conduct this systematic review.

### Eligibility Criteria

We included all controlled studies that compared stem cell therapy to placebo/vehicle or no-treatment *in vivo* models of ischemic stroke, in which the outcome was measured with neurological function score (NFS) and infarct size/infarct volume. To prevent bias, the inclusion criteria were prespecified as follows: (1) focal ischemic stroke, induced by transient middle cerebral artery occlusion (MCAO) or permanent MCAO, no restriction on animal species, as well as gender, age, weight, and sample size; (2) controlled studies with control group (receiving vehicle, saline, positive control drug, or no treatment) and experimental group (receiving allogeneic or autologous stem cell therapy), and there was no restriction on dosage, mode, and time of initial treatment; and (3) studies that have both the NFS and infarct size/infarct volume outcome measurement. The exclusion criteria were as follows: (1) other types of articles except animal experimental articles, including clinical articles, case reports, comments, reviews, abstracts, and *in vitro* studies; (2) non-focal cerebral ischemia model, such as global cerebral ischemia model, hypoxic–ischemic models or traumatic models; (3) non-single intervention, the administration for the experimental group was stem cell with another therapy or cell type; (4) non-controlled studies that lack a control group; (5) low-quality articles with quality scores lower than 5; and (6) published in other language except English.

### Information Sources

Three databases (PubMed, EMBASE, and Web of Science) were searched for relevant published articles that assessed the effect of stem cells in animal models of cerebral ischemia and were reported in English in peer-reviewed journals up to August 1, 2020. The reference lists of all selected publications were also used to identify additional eligible studies.

### Search Strategy

Studies of stem cells in animal models of cerebral ischemia were identified from three electronic databases (PubMed, EMBASE, and Web of Science). The keywords used in the search strategy were (stem cell OR stem OR multipotent OR mesenchymal OR cell therapy) AND (stroke OR cerebrovascular OR cerebral infarct OR cerebral ischemia/reperfusion OR middle cerebral artery OR middle cerebral artery occlusion). The Boolean (exact text) used in the search is in the Supplementary Material.

The publication time is from the inception of each database up to August 1, 2020. All searches were limited to studies on animals, published in English, and the stroke model was ischemic.

### Study Selection

The first selection was made using the following additional filters: “stem cell” and “stroke.” Then, a second selection was made from reading the titles and abstracts to assess if the content seemed to fit the inclusion criteria. Full text availability and criteria were verified before considering the inclusion of articles. The detailed research method was presented according to the PRISMA flow diagram.

### Data Extraction

The following details were extracted by two investigators from the included studies: (1) publication year and the first author’s name, type of ischemia (temporary or permanent); (2) characteristics of the animals used, including species, sex, and animal number per group; and (3) treatment information, including stem cells (donor species and tissue source), intervention regime (anesthetic, time for injections, method of administration, and number of cells injected). When a publication reported more than one experiment or where an experiment contained more than one individual comparison, they were regarded as independent experiments, and data for every individual comparison from each experiment were extracted, respectively. If the experimental group of animals received different doses of stem cell administration with a single control group, we used the data for the highest dose. If neurobehavioral tests were performed at different times, we only extracted data for the final time point reported. If the published data were missing or only expressed graphically, we contacted the authors for further information, and when a response was not received, we measured the numerical values from the graphs by using digital ruler software or exclude them. For each comparison, we extracted the data of mean value and standard deviation from each experimental and control group of every study.

### Risk of Bias Assessment

We assessed the methodological quality of the included studies by using the criteria according to a checklist as previously described (Macleod et al., 2004). These criteria were as follows: (1) peer-reviewed, which is an anonymized review process. The contribution will be initially assessed by the editor, and papers deemed suitable are then typically sent to a minimum of two independent expert reviewers to assess the quality of the paper; (2) statement of control of temperature; (3) random allocation to treatment or control; (4) blinded assessment of outcome; (5) use of anesthetic without significant intrinsic neuroprotective activity; (6) appropriate animal model which uses animals without relevant comorbidities (aged, diabetic, or hypertensive); (7) sample size calculation; (8) compliance with animal welfare regulations; and (9) statement of potential conflict of interests. Each item of the nine-item scale contributed one point, and each study was given a quality score. Two authors independently evaluated the methodological quality of the included articles. When one author thought that the quality score of one paper is higher than 5 and the other author thought that the score is lower than 5, we will discuss and consult with the corresponding author (G-QZ). The incidence of this situation is less than 1% because the two authors who evaluated the quality score have similar opinions basically.

### Summary Measures

The main outcome measurements were the NFS and infarct size/infarct volume. Neurological function was mainly examined with modified neurological severity score, which is a composite of motor, sensory, reflex, and balance tests, and the higher score shows more severe injury. The measurement of infarct volume was mainly through immunohistochemistry and triphenyltetrazolium chloride staining. A total of 49 of the included studies conducted immunohistochemistry, and the specific markers used in this article to evaluate cellular ischemia were different. Most articles (75.5%) used one or two. Among them, glial fibrillary acidic protein and NeuN were the most common markers, with 37 and 26 articles using them, respectively. In addition, there are only nine, eight, seven, and five articles using dual adrenocortical hormone, microtubule-associated protein 2, tubulin III, and neuron-specific enolase. The lesion volume was determined as follows: corrected infarct area = [infarct - (ipsilateral hemisphere - contralateral hemisphere)] / contralateral hemisphere × 100, as described previously (Swanson et al., 1990), in order to eliminate the overestimation of infarct size and the measurement difference between different methods.

### Synthesis of Results

Neurological function score and infarct volume were all considered as continuous data, and these outcome indicators conducted a global estimate of the combined effect sizes by calculating the standardized mean difference (SMD) or weighted mean difference (WMD) and 95% confidence intervals (CI) utilizing the random effects model. WMD is a standard statistic that measures the absolute difference between the mean values in two groups. It estimates the amount by which the experimental intervention changes the outcome on average compared with the control. It can be used as a summary statistic in meta-analysis when the outcome measurements in all studies are made on the same scale. SMD is used as a summary statistic in meta-analysis when the studies all assess the same outcome but measure the outcome in a variety of ways (Vesterinen et al., 2014).

### Risk of Bias Across Studies

Publication bias was assessed by using funnel plot and Egger’s test. The *I*^2^ statistic was used for the assessment of heterogeneity.

### Additional Analyses

To explore the impact of factors modifying the outcome measures, we conducted pre-stratified subgroup analyses according to the following variables: MCAO model induction, recipient species, recipient sex, donor species, anesthetic, number and type of cell used, time and route of administration, and manipulation of stem cells prior to implantation. Difference between groups was assessed by partitioning heterogeneity and using the *χ*^2^ distribution with *n*-1 degrees of freedom (df), where *n* equals the number of groups. Meta-regression analyses were conducted to search for systematic differences among study design characteristics that potentially explained the sources and extent of heterogeneity between studies (Guyatt et al., 2012). The meta-regression was univariate rather than multivariate, and we calculated adjusted *R*^2^ values to explain the proportion of the observed variability in the observed effect size for a group of experiments explained by variation in the independent variable in question (Antonic et al., 2013).

All statistical analyses were performed with RevMan version 5.3 and Stata version 15.0. Probability value *P* < 0.05 was considered significant.

## Results

### Study Selection

**FIGURE 1:**
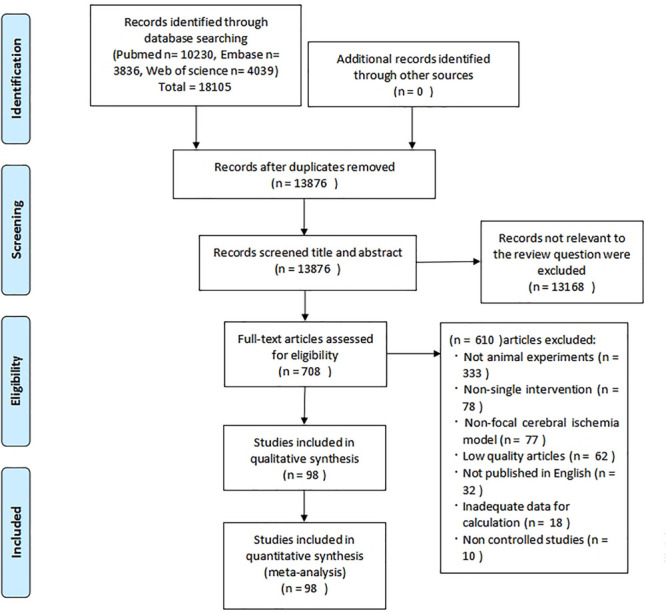
Flow diagram of the search process.

Electronic searching identified 18,105 potentially relevant records, of which 13,876 articles remained after the removal of duplicates. After going through the titles and abstracts, 13,168 papers were excluded for clearly irrelevant content. By reading the full text of the remaining 708 articles which reported the efficacy of stem cell in experimental ischemic stroke, we obtained the full papers of 178 publications and assessed these for eligibility. Of these, 18 studies were excluded due to inadequate data for the outcome calculation, and 62 articles were excluded because their quality scores did not reach 5. Ultimately, 98 eligible studies remained for this meta-analysis, which included 141 comparisons of NFS and infarct volume (Figure 1).

### Study Characteristics

The studies involved a total of 5,200 experimental subjects from two species: mouse (*n* = 352) and rats (*n* = 4,848). Eighty-four out of the 98 studies (85.7%) utilized temporary MCAO models, and 14 studies (14.3%) utilized permanent MCAO models. Isoflurane was used in 28 studies (28.6%), chloral hydrate in 19 studies (19.4%), and halothane in 15 studies (15.3%), which were the three most commonly used anesthetic. MSCs had been used in 57 studies (58.2%), followed by neural stem/progenitor cells (NSPCs) in 25 studies. The stem cells mostly used in these studies were passage three or four cells (38.1%), and more than half of the studies (67%) used Dulbecco’s modified Eagle’s medium for the proliferation of stem cells. The dose of stem cells injected ranges from 5 × 10^4^ to 1.2 × 10^7^, and the time of administration was mainly focused on 1 to 7 days after operation. Stereotaxic administration in 46 studies was the most frequently reported method of administration. The study characteristics are shown in Supplementary Table 1, and information about the passage that was used for the stem cells is in Supplementary Table 2.

### Risk of Bias Within Studies

The median quality score across the 98 studies was 5.8 (range, 5–8). Twenty-one studies failed to report whether there was temperature control. Random allocation to treatment group and blinded assessment of outcome were described in 72 and 58 studies, respectively. Nineteen studies had clearly said that there was no use of anesthetic with significant intrinsic neuroprotective activity. Only one study calculated the sample size necessary to achieve sufficient power. The declaration of compliance with animal welfare regulations and the potential conflict of interests were described in 85 and 54 studies, respectively. Other items, such as if it were peer-reviewed and the use of an appropriate animal model, which are animals without relevant comorbidities, were well reported in all these studies. The methodological quality of each study is summarized in Supplementary Table 3.

### Meta-Analysis

**FIGURE 2:**
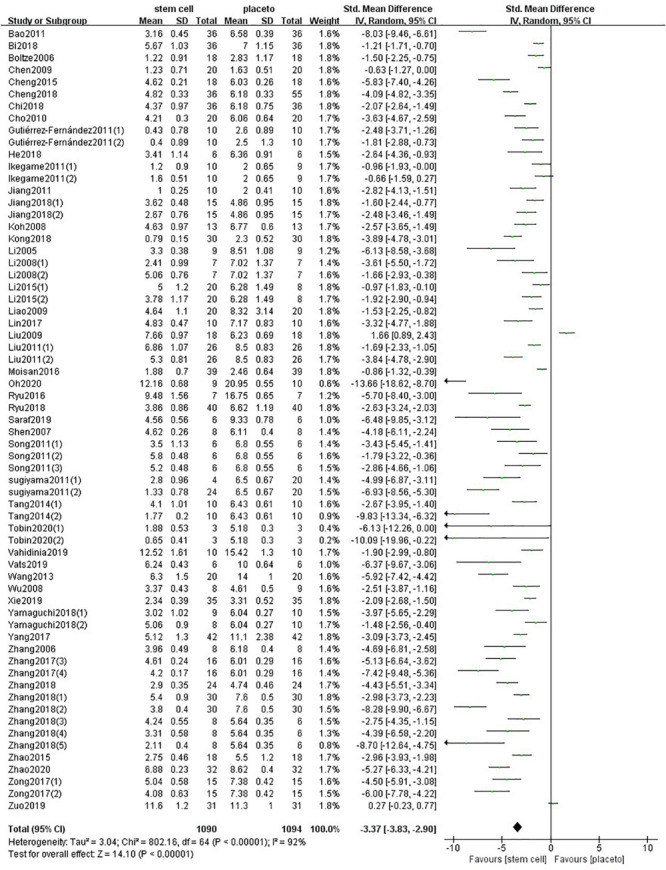
Summary of the data included in the meta-analysis of the use of stem cells to treat ischemic stroke with individual comparisons ranked according to their effect on neurological function.

**FIGURE 3:**
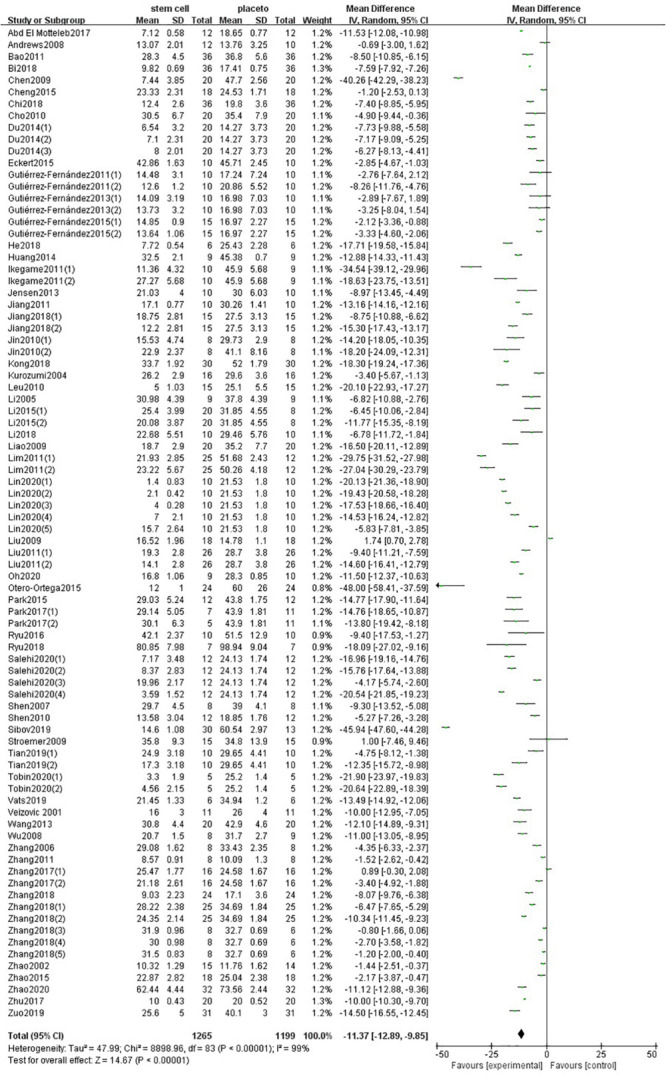
Summary of the data included in the meta-analysis of the use of stem cells to treat ischemic stroke with individual comparisons ranked according to their effect on infarct volume.

One hundred forty-one comparisons of 98 included studies (Veizovic et al., 2001; Zhao et al., 2002, 2015, 2018; Kurozumi et al., 2004; Li et al., 2005, 2008, 2018, 2016; Boltze et al., 2006; Zhang et al., 2006, 2011; Shen et al., 2007, 2010; Andrews et al., 2008; Koh et al., 2008; Kamiya et al., 2008; Wu et al., 2008, 2015; Chen et al., 2009; Hicks et al., 2009; Liao et al., 2009; Stroemer et al., 2009; Cho et al., 2010; Jin et al., 2010; Leu et al., 2010; Bao et al., 2011; Lim et al., 2011; Gutiérrez-Fernández et al., 2011, 2013, 2015; Ikegame et al., 2011; Jiang et al., 2011, 2019; Liu et al., 2009, 2011; Song et al., 2011; Sugiyama et al., 2011; Inoue et al., 2013; Jensen et al., 2013; Wang H. et al., 2013; Du et al., 2014; Huang et al., 2014, 2018; Mitkari et al., 2014; Tang et al., 2014; Cheng et al., 2015, 2018; Hosseini et al., 2015a,c; Ma et al., 2015; Otero-Ortega et al., 2015; Eckert et al., 2015; Park et al., 2015, 2017; Yang et al., 2015, 2018, 2017; Wang et al., 2015; Bacigaluppi et al., 2016; Choi et al., 2016; Moisan et al., 2016; Ryu et al., 2016, 2019; Hou et al., 2017; Lin et al., 2017, 2020; Souza et al., 2017; Yamashita et al., 2017; Zhang J. J et al., 2017; Zhang T. et al., 2017; Zong et al., 2017; Zhu et al., 2017; Abd El Motteleb et al., 2018; Bi et al., 2018; Chi et al., 2018; Kong et al., 2018; Nito et al., 2018; Sowa et al., 2018; Yamaguchi et al., 2018; Yu et al., 2018; Zhang H.L. et al., 2018; Zhang G. et al., 2018; Cherkashova et al., 2019; He et al., 2019; Saraf et al., 2019; Sibov et al., 2019; Son et al., 2019; Tian et al., 2019; Vahidinia et al., 2019; Vats et al., 2019; Xie et al., 2019; Zuo et al., 2019; Lam et al., 2020; Salehi et al., 2020; Tobin et al., 2020; Paudyal et al., 2020; Oh et al., 2020) involving 5,200 animals examined the effect of stem cell transplantation on the neurological function and the infarct volume in animal models for stroke. The pooled analysis indicated that the animals in the treatment group significantly improved in neurological function more than the animals in the control group (SMD = −3.37, 95% CI −3.83 to −2.90, *P* < 0.00001); heterogeneity: chi^2^ = 802.16, *df* = 64 (*P* < 0.00001), *I*^2^ = 92% (Figure 2). The pooled analysis indicated that the animals in the treatment group significantly improved in infarct volume more than the animals in the control group (WMD = −11.37, 95% CI -12.89 to −9.85, *P* < 0.00001); heterogeneity: chi^2^ = 8898.96, *df* = 83 (*P* < 0.00001), *I*^2^ = 99% (Figure 3).

### Stratified Meta-Analysis

In the stratified meta-analysis, the impact of study characteristics on the effect sizes was examined.

For neurological function, the stratified analysis showed that significant differences in effect sizes were observed relative to donor species (*P* < 0.00001) and time of administration (*P* = 0.0003). No significant differences in effect sizes were observed relative to the type of ischemia (*P* = 0.13), recipient species (*P* = 0.22), recipient sex (*P* = 0.95), anesthetic (*P* = 0.23), stem cell (*P* = 0.59), cell manipulations (*P* = 0.39), dose range (*P* = 0.09), and method of administration (*P* = 0.16) (Supplementary Table 4).

For infarct volume, the stratified analysis showed that significant differences in effect sizes were observed relative to the type of ischemia (*P* = 0.001), recipient sex (*P* < 0.00001), anesthetic (*P* < 0.0001), time of administration (*P* = 0.03), and method of administration (*P* < 0.00001). No significant differences in effect sizes were observed relative to recipient species (*P* = 0.25), donor species (*P* = 0.36), stem cell (*P* = 0.47), cell manipulations (*P* = 0.55), and dose range (*P* = 0.54) (Supplementary Table 5).

**FIGURE 4:**
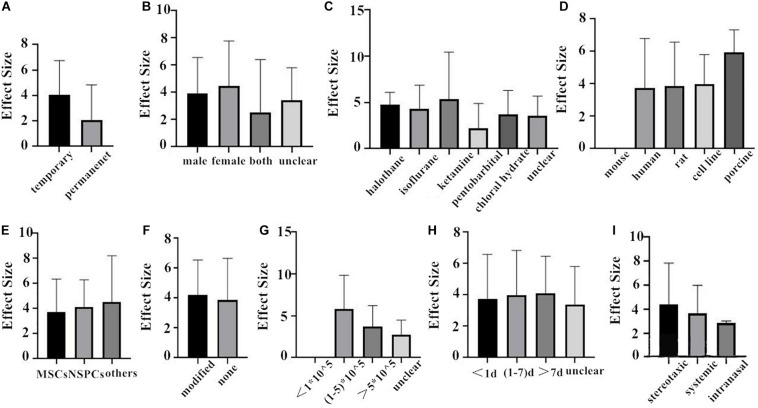
The impact of study characteristics on the effect sizes aimed at rat. **(A)** Type of ischemia, **(B)** sex of animals, **(C)** anesthetic used, **(D)** donor species, **(E)** stem cell, **(F)** cell manipulations, **(G)** dose range, **(H)** time of administration, and **(I)** route of delivery.

**FIGURE 5:**
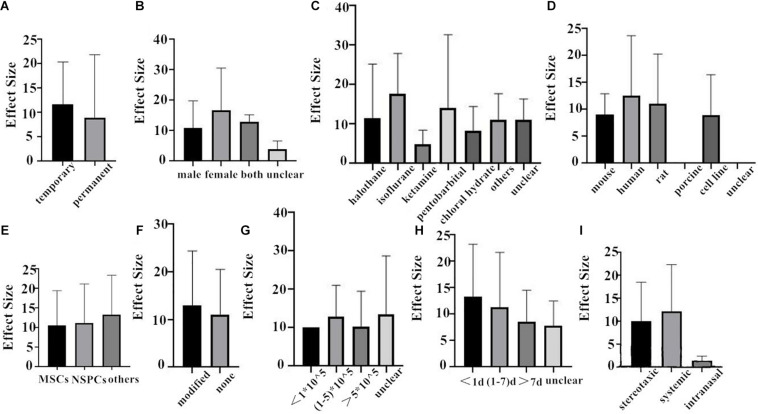
The impact of study characteristics on the effect sizes aimed at rat. **(A)** Type of ischemia, **(B)** sex of animals, **(C)** anesthetic used, **(D)** donor species, **(E)** stem cell, **(F)** cell manipulations, **(G)** dose range, **(H)** time of administration, and **(I)** route of delivery.

First of all, we do the subgroup analysis aimed at rat. The effect size of the transient MCAO model was larger than that of the permanent MCAO model both in NFS and infarct volume (Figures 4A, 5A). It may be that persistent cerebral ischemia causes lasting damage to rat cerebral tissue. Similarly, sex with efficacy was higher in females, whether it is in NFS or infarct volume (Figures 4B, 5B), which showed that female rats recovered better after stroke. Besides that, as for anesthetic, the effect size of halothane was larger, while the situation was different in the infarct volume (Figures 4C, 5C). The result means that the use of halothane can better improve the neurological deficit, and the administration of isoflurane can do better in decreasing the infarct volume. This may be related to the intrinsic neuroprotective activity of these two anesthetics. Porcine as a source of stem cells was associated with substantial improvement in NFS, and human stem cells are better at decreasing the infarct volume (Figures 4D, 5D). Meanwhile, higher estimates of effect size by NSPCs were observed in NFS, while efficacy was higher for other types of stem cell in infarct volume (Figures 4E, 5E). A higher effect size of gene-modification cells in rat is shown, which is similar to the reported neuroprotective effects of gene-modification cells in the literatures (Figures 4F, 5F), even though the degree of variance is not high. In addition, a moderate dose of stem cells (1–5 × 10^5^) can better improve the neurological deficit, but which certain dose is better for reducing infarct volume is unclear (Figures 4G, 5G). It is better to give stem cell therapy as early as possible after stroke for efficacy was observed to be higher within 1 day after stroke in infarct volume, and the effect size was similar to the group administrated at 7 days after stroke in NFS (Figures 4H, 5H). Moreover, stereotaxic injection was more effective in NFS and got very close to systemic administration in improving infarct volume outcome (Figures 4I, 5I).

Besides that, the subgroup analysis aimed at mice cannot be carried out because the date related to mice is not adequate enough.

**FIGURE 6:**
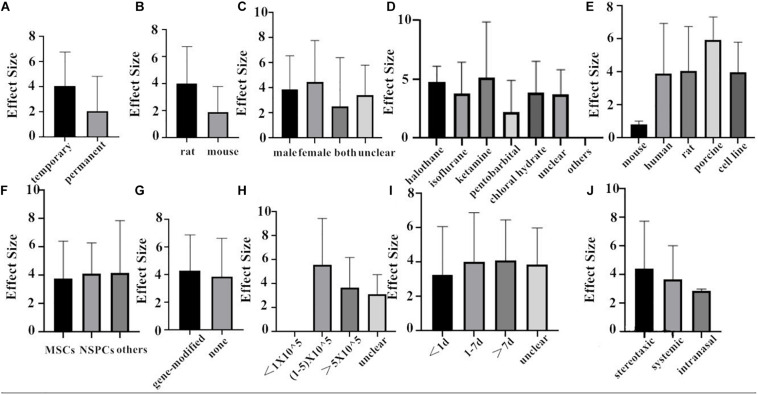
The impact of study characteristics on the effect sizes of two species. **(A)** Type of ischemia, **(B)** species, **(C)** sex of animals, **(D)** anesthetic used, **(E)** donor species, **(F)** stem cell, **(G)** cell manipulations, **(H)** dose range, **(I)** time of administration, and **(J)** route of delivery.

**FIGURE 7:**
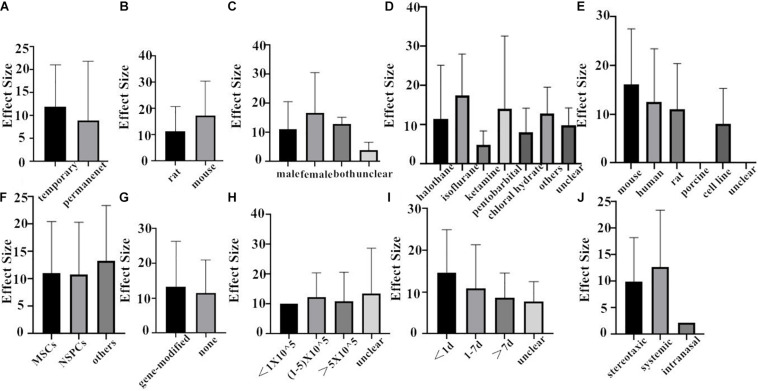
The impact of study characteristics on the effect sizes of two species. **(A)** Type of ischemia, **(B)** species, **(C)** sex of animals, **(D)** anesthetic used, **(E)** donor species, **(F)** stem cell, **(G)** cell manipulations, **(H)** dose range, **(I)** time of administration, and **(J)** route of delivery.

Finally, we combined the data from the two species and conducted the subgroup analysis. In the analysis for the outcome measure according to NFS, the effect size of transient MCAO model was larger than that of the permanent MCAO model, and the situation was the same in infarct volume (Figures 6A, 7A). The results illustrated that the transient MCAO model could improve the prognosis of stroke more effectively. Similarly, sex with efficacy was higher in males compared with females both in NFS and infarct volume (Figures 6C, 7C). It seems that males will recover better than females, but it seems different when concerning recipient species. Compared with mouse, rat was less effective at improving infarct volume but more effective in improving NFS (Figures 6B, 7B). The use of halothane at MCAO induction was associated with a substantial improvement in NFS, while as for infarct volume, isoflurane seems better (Figures 6D, 7D). This may be related to the intrinsic neuroprotective activity of these two anesthetics. Porcine, as a source of stem cells, was with substantial improvement in NFS, while mouse seems better in decreasing infarct volume (Figures 6E, 7E). Meanwhile, in the subgroup analysis for the outcome of the type of stem cell, efficacy was higher for NSPCs in NFS, while the other types of stem cell such as MHP36 cells and SP cells had higher estimates of effect size in infarct volume (Figures 6F, 7F). A higher effect size of gene-modification cells in rat is shown, which is similar to the reported neuroprotective effects of gene-modification cells in the literatures (Figures 6G, 7G), even though the degree of variance is not high. In addition, the effect size was observed to be larger with the dose of 1–5 × 10^5^ in NFS and the unclear dose of stem cell in infarct volume (Figures 6H, 7H). It illustrated that the injection of stem cells at a moderate dose could help reduce the neurological deficit effectively. As for the subgroup of the unclear dose, it is a group wherein the articles did not explicitly mention the dose of treatment, so we classified all of these articles into the unclear dose group. Improvement was seen in the stem cell therapy that was administrated at 7 days after cerebral ischemia in NFS, while in infarct volume, it was clear when the administration was done within 1 day after stroke (Figures 6I, 7I). It means that stem cell therapy in the acute phase (24 h–7 days) can better rescue the neurons in the ischemic penumbra of the brain. Moreover, stereotaxic injection was more effective in NFS and got very close to systemic administration in improving infarct volume outcome (Figures 6J, 7J).

### Meta-Regression

To further explore heterogeneity among the studies, meta-regression was conducted to investigate the effect of characteristics on neurological function and infarct volume.

For neurological function, dose range (*P* = 0.045) was a significant source of heterogeneity, while type of ischemia (*P* = 0.057), recipient species (*P* = 0.108), recipient sex (*P* = 0.959), anesthetic (*P* = 0.154), donor species (*P* = 0.189), stem cell (*P* = 0.76), cell manipulations (*P* = 0.582), time of administration (*P* = 0.315), and method of administration (*P* = 0.83) had little effect on heterogeneity (Supplementary Table 4).

For infarct volume, we found that anesthetic (*P* = 0.028) and time of administration (*P* = 0.024) accounted for a significant proportion of the between-study heterogeneity in studies, while type of ischemia (*P* = 0.243), recipient species (*P* = 0.852), recipient sex (*P* = 0.44), donor species (*P* = 0.097), stem cell (*P* = 0.623), cell manipulations (*P* = 0.844), dose range (*P* = 0.884), and method of administration (*P* = 0.923) had little to do with heterogeneity (Supplementary Table 5).

### Publication Bias

**FIGURE 8:**
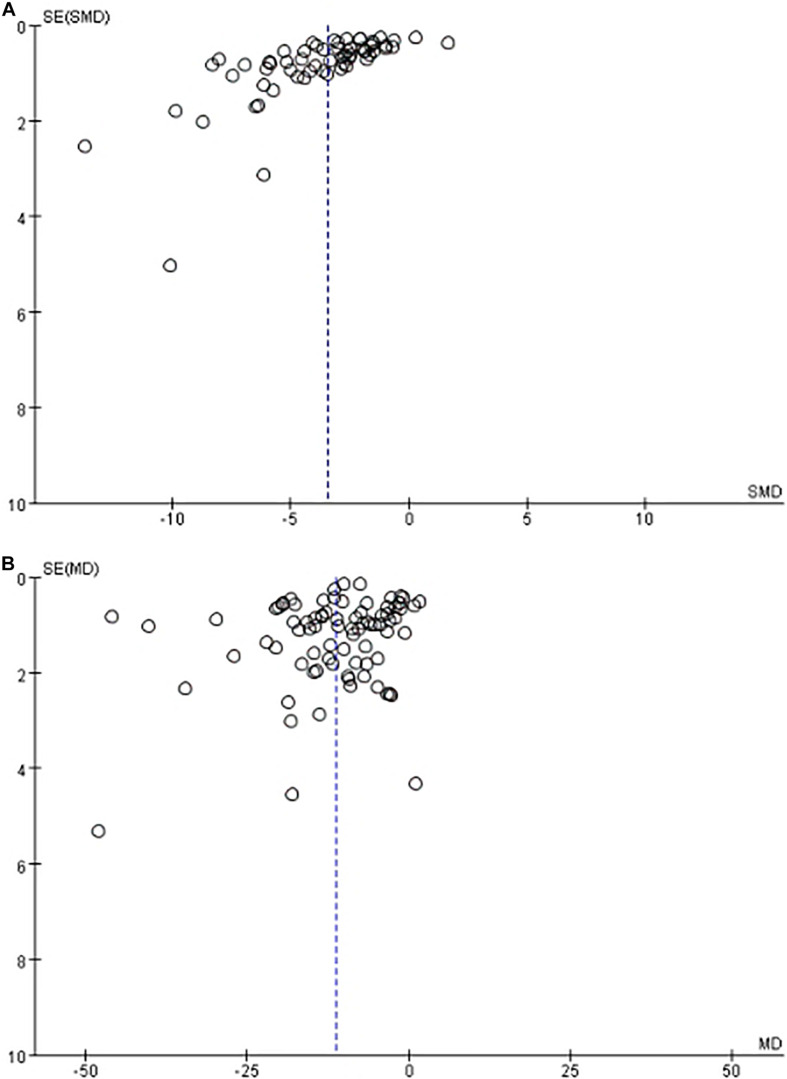
The funnel plot of the effect of stem cell on neurological function **(A)** and infarct volume **(B)** after experimental ischemic stroke.

**FIGURE 9:**
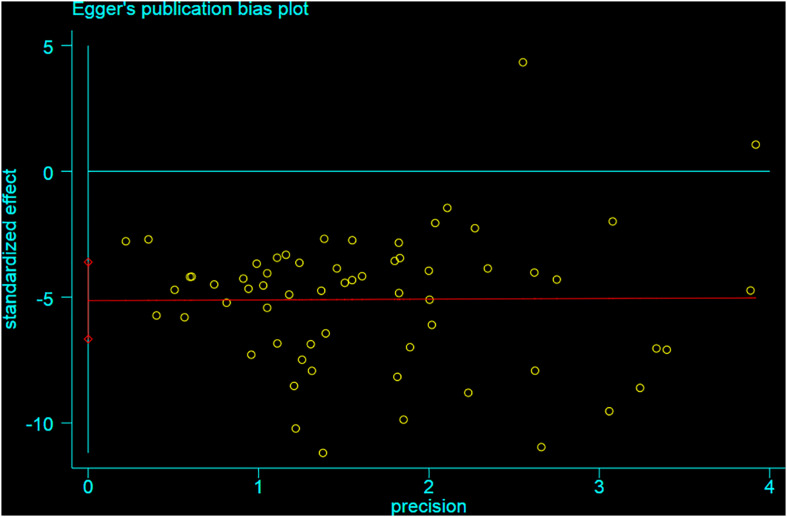
Egger test for the assessment of the statistical significance of publication bias of neurological function.

**FIGURE 10:**
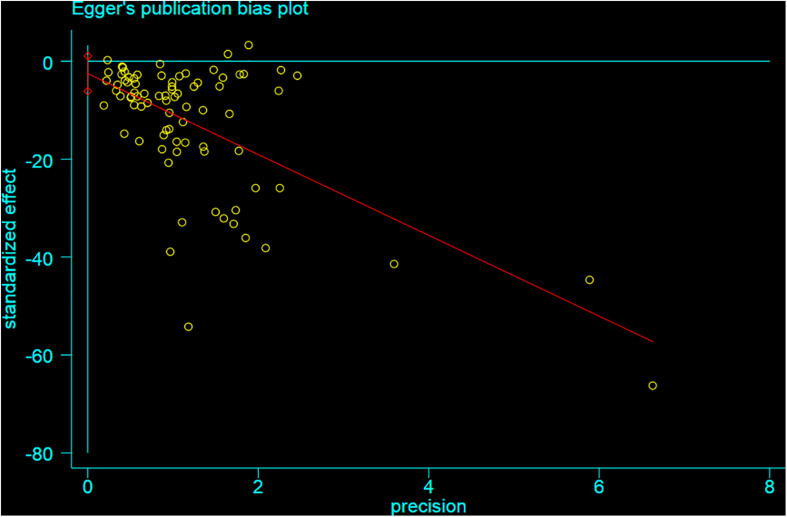
Egger test for the assessment of the statistical significance of publication bias of infarct volume.

Funnel plot is a method to identify publication bias or other bias and judge whether there is bias in meta-analysis according to the degree of asymmetry. It (Figure 8) showed asymmetry, indicating a potential publication bias. Egger test is a statistical test of funnel plot asymmetry to determine whether publication bias or other bias has statistical significance. Through Egger’s test, the publication not existed in neurological function data (Figure 9, *p* < 0.163), but in infarct volume data (Figure 10, *p* = 0.001).

## Discussion

### Efficacy of Stem Cell-Based Therapy

The present study demonstrated that stem cell-based therapy improved neurological deficits and reduced infarct volume, reinforcing the evidence for a neuroprotective role of stem cell therapy in experimental ischemic stroke. However, our summary estimates should be considered as the average efficacy rather than the best estimate of a single “true” efficacy (Zhao et al., 2002) because of the existence of heterogeneity.

### Methodological Considerations

In the present study, the overall quality of the included studies was relatively high. Thus, to some extent, the global estimated effect may be guaranteed because there were relatively few articles that did not use the blind method, did not randomly evaluate the result, or did not report the number of exits, which are key elements in the quality assessment of preclinical studies (Wardlaw et al., 2000). In addition, as a previous study once discussed (Macleod et al., 2004), the quality of study design is an important factor affecting the outcome. Especially when such trials are included in systematic reviews, the improvement of methodological quality will reduce bias (Xie et al., 2015).

### Stratified Analysis

We found that the studies which used the transient MCAO model showed larger behavioral gains as well as infarct volume. The transient MCAO is also the most widely used animal models for experimental ischemic stroke and well simulates actual pathological conditions after thrombolysis in clinical practice. The recovery of cerebral blood flow plays a role on the improvement of neurological function, but the cerebral parenchyma was sensitive to reperfusion injury in the meantime, so there was no significant improvement in the infarct volume in a short experimental observation time.

Compared with mouse, rat was less effective at improving infarct volume but more effective in improving NFS. However, these accounted for a small proportion of the overall dataset, so these findings should be interpreted with caution. Meanwhile, the use of different types of anesthetic at MCAO induction was associated with recovery. In terms of neurological function score, the use of halothane showed a substantial improvement. For infarct volume, studies using isoflurane led to higher estimates of effect, possibly associated with its significant intrinsic neuroprotective activity (Macleod et al., 2004).

The most widely used stem cells in the studies were MSCs, but in the overall analysis, NSPCs were associated with a substantially larger effect in NFS, while other types of stem cells such as MHP36 cells and SP cells showed a higher improvement in infarct volume. These contrasts were less obvious in the effect size of manipulation of modified stem cell even though previous studies demonstrated that gene-modified stem cell can increase cell survival and enhance their function in cell therapy (Phillips and Tang, 2012). Studies that used these genetically modified stem cells do not show an obvious high estimate of effect in neurological function and infarct volume. Zhao et al. (2010) found increasing benefits from concentrations of as low as 10,000 implanted stem cells, while we see that the dose that significantly eased the neurological function recovery is 1–5 × 10^5^. When concerning the decrease of infarct volume, it seems to be the abnormally high effect sizes reported in studies where the dose range was unclear. However, it should be interpreted with caution because these results are based on a small number of comparisons.

The optimal time and the best route for cell transplantation against ischemic stroke have always been the focus of controversy. Our results suggested that stem cell therapy within 24 h after cerebral ischemia can decrease the volume, and the administration that was employed at 7 days after ischemia can improve the function, which is only partly consistent with what Hosseini et al. (2015b) had reported. In addition, we found that the effect of three different methods of treatment was similar in promoting the recovery of neurological function, but in infarct volume, systemic delivery brought a higher efficacy than the way of stereotaxic and intranasal administration. Intranasal administration was actually *via* a specific site, the olfactory region, thus bypassing the blood–brain barrier and delivering therapeutic agents directly to the central nervous system (Illum, 2000; Thorne and Frey, 2001). Although it acts directly on brain tissue, its effect is not as good as expected. Given the size of the stem cell itself, the underlying feasibility and following therapeutic effect is worth investigating in greater depth in the future.

### Limitations of Our Study

Firstly, our search strategy was only able to include the majority of published studies in three English databases; other languages and unpublished studies were not taken into account, which may lead to a certain degree of selective bias (Guyatt et al., 2011). Secondly, there are many methods to build a stroke induction model, such as suture method, electric coagulation, photothrombosis, microembolism, thermocoagulation, and so on. Among the final studies included, there are more articles using rat to induce a stroke model, where suture method is more effective and convenient, so there are fewer articles using other methods. Therefore, our results may not be able to comprehensively assess the impact of various methods of stroke induction on the outcome measurements. Thirdly, analytic data in our meta-analysis were not available, such that of unpublished information of both study quality and study design features (the sex of a cohort of animals or the route of cell implantation); we have either analyzed that as unclear or inferred that these things that were not reported did not occur. Besides that, we present a series of univariate analyses; meta-regression might provide more robust insights, but these techniques are not well established (Zhao et al., 2002). Furthermore, our research is observational rather than experimental, so we can only report associations rather than causation. Finally, we limited our analysis to studies that have both neurological function and infarct volume outcome measures. Thus, we will disregard other benefits seen in either of them as a main outcome indicator.

## Conclusion

The present finding indicated that stem cell provided statistically significant benefits for stroke whether in neurological function or infarct volume. Therefore, the findings of the present systematic review, at least to a certain extent, provided supporting evidence for the future use of stem cell for stroke. However, the validity of these positive findings should be interpreted with caution due to some methodological limitations.

Besides that, even though the result we yielded is promising, certain safety concerns must also be properly addressed for its further use in the clinic in the future. First of all, tumor formation has been reported with the transplantation of stem cells. For example, one study declared that mice iPSCs expanded and formed much larger tumors in mice post-ischemic brain than in sham-operated brain until 28 days after transplantation (Kawai et al., 2010), but in a clinical trial conducted by Honmou et al. (2011) the safety of transplantation was shown. After 1 year of treatment, 12 patients who received stem cell therapy showed no adverse reactions such as central nervous system tumor, abnormal cell growth, venous thromboembolism, systemic malignant tumor, or systemic infection (Honmou et al., 2011). Therefore, before stem cells are allowed to be extensively used in the clinic, tumorigenesis is one of the next research points. In addition, whether the application of exogenous stem cell will cause immunological rejection and the ethical problem it brings also need to be paid attention to. Even though the current preclinical data is positive, studies of stem cell after stroke in humans thus far have focused on autologous cells, and so trials examining the safety of allogeneic stem cell in humans are needed.

On the other hand, there are NSPCs that existed in the subventricular and dentate gyrus zone of the brain. During ischemia, the proliferation and differentiation of endogenous neural stem cells is not sufficient enough to induce neural repair, which could contribute to the permanent disability of stroke patients (Bond et al., 2015). Thus, whether we can promote the proliferation and differentiation of stem cells in the brain through some exogenous interventions to improve the prognosis of stroke may be a novel therapeutic approach. Furthermore, whether we can promote the proliferation of endogenous stem cells through some exogenous interventions may also be a novel therapeutic approach. For example, Zhang et al. (2020) showed that treatment with artesunate promoted the proliferation of NSPCs *via* some molecular mechanisms, subsequently reducing the infracted brain volume and alleviating motor function impairment caused by MCAO. Zhuang et al. (2012) had undertaken a study to examine the promotive effect of salvianolic acid B (Sal B) on NSPC proliferation and neurogenesis. The result showed that Sal B was capable of promoting the proliferation of NSPC and neurogenesis *via* the PI3K/Akt signaling pathway. These findings suggested that it is possible that we could promote the recovery of stroke *via* endogenous stem cells. Besides that, the application of endogenous stem cells will avoid the possibility of tumorigenesis, immunological rejection, ethical problems, and other adverse reactions.

In addition, compared with some previous systematic reviews on stem cells for stroke, our review is more innovative. First of all, previous reviews on stem cell therapy for stroke are mostly about MSCs. We included studies of all kinds of stem cells and analyzed the different effects of different types of stem cell on neurological deficit and infarct volume. Secondly, through the assessment of the methodological quality of the literature, we only included studies whose quality score is higher than the average level in order to make the results more reliable and convincing. Finally, while other studies only discuss the effect of stem cell therapy on neurological deficit, our paper selected two outcome indicators—neurological function and infarct volume—and we are eager to make a more comprehensive assessment of stem cells on ischemic stroke.

## Data Availability Statement

The original contributions presented in the study are included in the article/Supplementary Material, further inquiries can be directed to the corresponding author/s.

## Author Contributions

X-LZ, X-GZ, Y-RH, Y-YZ, P-JY, X-JZ, XL, and Y-JW analyzed the data and carried out the statistical analysis. G-QZ acted as an arbitrator in the review. G-QZ, X-LZ, and X-GZ conceived and designed the article, supervised the study, and contributed to finalize the manuscript. All authors participated in the study design, searched the databases, extracted and assessed the studies, and drafted the manuscript and reviewed the manuscript.

## Conflict of Interest

The authors declare that the research was conducted in the absence of any commercial or financial relationships that could be construed as a potential conflict of interest.
