# Supplementary material for: Stem Cell-Based Therapy for Experimental Ischemic Stroke: A Preclinical Systematic Review
Source: Front Cell Neurosci. 2021 Apr 14;15:628908. doi: 10.3389/fncel.2021.628908 (PMC8079818; doi:10.3389/fncel.2021.628908)
Supplement: Supplementary file 1 [file Data_Sheet_1.docx]

**Table S1: Study Characteristics of included studies**

| Study  (years) | Type of Ischemia | Species | Total No. | Sex | Anaesthetic | Donor species | Type of stem cell | Dose Range | Time of admin (mins) | Method of administration |
| --- | --- | --- | --- | --- | --- | --- | --- | --- | --- | --- |
| Choi2016[1] | Temporary | mouse | 15 | male | tiletamine | human | BMSCs | 1.0×10^5^ | 1440 | systematic(IV) |
| Jiang2018[2] | Temporary | rat | 60 | male | chloral hydrate | rat | BMSCs | unclear | 0 | systematic(IV) |
| Zhao2020[3] | Temporary | rat | 160 | both | chloral hydrate | rat | ADSCs | 2.0×10^6^ | unclear | systematic(IV) |
| Tian2019[4] | Temporary | rat | 30 | male | chloral hydrate | mouse | NSCs  (modified) | 5.0×10^6^ | 360 | systematic(IV) |
| Zhang2011 [5] | Temporary | rat | 48 | unclear | chloral hydrate | rat | NSCs | 1.0 ×10^6^ | unclear | stereotaxic |
| Inoue2013 [6] | Permanent | rat  rat | 14  10 | male  male | isoflurane  isoflurane | human  human | SHED  BMMSCs | unclear  unclear | 4320-21600  4320-21600 | intranasal  intranasal |
| Du2014[7] | Temporary | rat | 100 | male | chloral hydrate | rat | ADSCs | 5.0×10^5^ | 1440 | systematic(IV)  systematic(IA)  stereotaxic |
| Liu2009[8] | Permanent | rat | 72 | female | chloral hydrate | rat | MSCs | 1.0×10^6^ | 1440 | stereotaxic |
| Tobin2020[9] | Temporary | rat | 12 | female | isoflurane | rat | MSCs | 1.25×10^6^ | 180 | systematic(IV) |
| Zong2017[10] | Temporary | rat | 90 | male | chloral hydrate | rat | BMSCs  (modified) | 1.0×10^6^ | 1440 | stereotaxic |
| He2018[11] | Temporary | rat | 21 | male | chloral hydrate | rat | BMSCs | 2.0×10^6^ | 720 | systematic(IV) |
| Nito2018[12] | Temporary | rat | unclear | male | halothane | human | DPSCs | 1.0×10^6^ | 0  180 | systematic(IV) |
| Zhang2017[13] | Temporary | rat | 48 | male | chloral hydrate | human | NSCs  (modified) | 5.0×10^6^ | 1440 | systematic(IV) |
| Souza2017[14] | Temporary | rat | 16 | female | ketamine | unclear | BMMCs | 1.0×10^6^ | 1440 | stereotaxic |
| Li2015[15] | Temporary | rat | 48 | male | chloral hydrate | rat | BMMCs | 1.0×10^7^ | 1440 | systematic(IV) |
| Son2019[16] | Temporary | rat | 40 | male | isoflurane | human | MSCs | 2.0×10^5^ | unclear | stereotaxic |
| Huang2018[17] | Temporary | rat | unclear | male | isoflurane | unclear | MSCs  (modified) | 2.0×10^6^ | 1440 | systematic(IV) |
| Zhang2018[18] | Temporary | rat | 42 | male | chloral hydrate | rat | BMSCs | 3.0×10^6^ | 10080 | intranasal  systematic(IV)  systematic(IA) |
| Yang2015[19] | Temporary | rat | 24 | unclear | unclear | rat | BMSCs | 5.0×10^6^ | 4320 | systematic(IV) |
| Liu2011[20] | Temporary | rat | 78 | male | chloral hydrate | rat | BMSCs  (modified) | 3.0×10^6^ | 1440 | systematic(IV) |
| Zhang2006 [21] | Temporary | rat | 16 | male | halothane | rat | BMSCs | 2.0×10^6^ | 1440 | systematic(IA) |
| Shen2007 [22] | Temporary | rat | 25 | female | halothane | rat | MSCs | 3.0×10^6^ | unclear | systematic(IV) |
| Koh2008 [23] | Temporary | rat | 36 | unclear | pentobarbital | human | UCMSCs | 6.0×10^5^ | 20160 | stereotaxic |
| Lim2011[24] | Temporary | rat | 74 | male | isoflurane | human | UCMSCs | 1.0 ×10^6^ | 4320 | systematic(IV) |
| Bao2011[25] | Temporary | rat | 72 | male | chloral hydrate | human | BMSCs | 5.0×10^5^ | 4320 | stereotaxic |
| Ikegame2011[26] | Temporary | mouse  mouse | 19  19 | male  male | isoflurane isoflurane | mouse  mouse | BMSCs  ADSCs | 1.0 ×10^6^  1.0 ×10^6^ | 90  90 | systematic(IV)  systematic(IV) |
| Song2011 [27] | Temporary | rat | 24 | male | unclear | cell line | NSCs | 4.0 ×10^6^ | 1440-10080 | systematic(IV) |
| sugiyama2011[28] | Temporary | rat | 48 | male | isoflurane | porcine | SP cells | 1.0 ×10^6^ | 1440 | stereotaxic |
| Wang2013 [29] | Temporary | rat | 40 | male | halothane | cell line | MSCs | unclear | 1440 | systematic(IV) |
| Jensen2013 [30] | Temporary | rat | 20 | male | isoflurane | cell line | NSCs | 2.5×10^5^ | 10080 | stereotaxic |
| Cheng2015 [31] | Temporary | rat | 36 | male | pentobarbital | cell line | NSCs | 5.0×10^6^ | 1440 | systematic(IV) |
| Boltze2006 [32] | Permanent | rat | 36 | unclear | ketamine | human | UCBCs | 1.0×10^6^ | 1440 | systematic(IV) |
| Cho2010[33] | Temporary | rat | 134 | male | unclear | human | MSCs  (modified) | 6.0×10^5^ | 20160 | stereotaxic |
| Lapi2015 [34] | Temporary | rat | 182 | male | alpha-chloralose | rat | BMSCs | 1.0×10^6^ | 120 | systematic(IA) |
| Lam2020[35] | Temporary | rat | 60 | female | ketamine | rat | MSCs | 8.0×10^5^ | 1440 | stereotaxic |
| Yamaguchi2018[36] | Temporary | rat | 58 | male | unclear | human | MSCs | 1.0×10^6^ | 1440 | systematic(IA) |
| Gutiérrez-Fernández2015[37] | Permanent | Rat  rat | 30  30 | male  male | ketamine  ketamine | Human  rat | ADMSCs  ADMSCs | 2.0×10^6^ | unclear | systematic(IV) |
| Zhang2017[38] | Temporary | mouse | unclear | male | ketamine | mouse | NSCs | 1.0×10^6^ | 1440 | stereotaxic |
| Lin2020[39] | Temporary | rat | 70 | male | isoflurane | rat | EPCs | 1.2×10^6^ | 180  4320  10080  20160 | systematic(IA) |
| Salehi2020[40] | Temporary | rat  rat | 72  72 | male  male | chloral hydrate  chloral hydrate | rat  rat | NSCs  BMSCs | 2.0×10^6^  2.0×10^6^ | 0  0 | systematic(IV)  systematic(IA) |
| Kong2018[41] | Temporary | rat | 60 | male | isoflurane | human | PDMCs  (modified) | 1.0×10^5^ | 1440 | stereotaxic |
| Yang2018[42] | Permanent | rat | 90 | male | isoflurane | rat | BMMCs | 3.0×10^6^ | unclear | systematic(IV) |
| Zhu2017[43] | Temporary | rat | 80 | male | chloral hydrate | rat | NSCs  (modified) | 5.0×10^5^ | 4320 | stereotaxic |
| Bacigaluppi2016[44] | Temporary | mouse | unclear | male | isofluorane | mouse | NPCs | 1.0×10^6^ | 4320 | systematic(IV) |
| Li2018[45] | Permanent | rat | 90 | male | isoflurane | rat | BMSCs | 1.0×10^6^ | unclear | systematic(IV) |
| Sowa2018[46] | Temporary | rat | 151 | male | halothane | human | DPSCs  (modified) | 1.0×10^6^ | unclear | systematic(IV) |
| Paudyal2020[47] | Temporary | rat | 40 | male | isoflurane | human | ADMSCs | 1.0×10^6^ | unclear | stereotaxic |
| Bi2018[48] | Temporary | rat | 180 | male | unclear | rat | BMSCs | 1.0×10^6^ | 1440 | stereotaxic |
| Ryu2018[49] | Temporary | rat | 40 | male | isoflurane | rat | ADMSCs | unclear | 2880 | stereotaxic |
| Wang2015[50] | Temporary | mouse | unclear | male | isoflurane | unclear | NSCs  (modified) | 5.0×10^4^ | 120 | stereotaxic |
| Yang2017[51] | Temporary | rat | 126 | male | isoflurane | human | MAPCs | 1.2×10^7^ | 1440 | systematic(IV) |
| Zhang2018[52] | Temporary | rat | 120 | male | chloral hydrate | rat | NSCs | 8.0×10^5^ | unclear | stereotaxic |
| Moisan2016[53] | Temporary | rat | 78 | male | unclear | human | BMSCs | unclear | unclear | systematic(IV) |
| Zhao2002[54] | Permanent | rat | 41 | male | methohexital sodium | human | BMSCs | unclear | 10080 | stereotaxic |
| Kurozumi2004[55] | Temporary | rat | 64 | unclear | halothane | human | BMSCs  (modified) | 5.0×10^5^ | 1440 | stereotaxic |
| Li2005[56] | Permanent | rat | 18 | unclear | halothane | rat | MSCs | 3.0×10^6^ | 10080 | systematic(IV) |
| Wu2008[57] | Temporary | rat | 23 | female | isoﬂurane | rat | BMSCs | 3.0×10^6^ | 1440 | systematic(IV) |
| Andrews2008[58] | Temporary | rat | 22 | male | pentobarbital | human | BMSCs | 1.5×10^6^ | 10080 | stereotaxic |
| Li2008[59] | Temporary | rat | 28 | male | isoflurane | human | MSCs | 1.0×10^6^ | 1440 | systematic(IV) |
| Liao2009 [60] | Temporary | rat | 50 | male | chloral hydrate | human | UCMSCs | 2.0×10^5^ | 1440 | stereotaxic |
| Chen2009 [61] | Temporary | rat | 75 | male | pentobarbital | rat | NSCs  (modified) | 5.0×10^5^ | 4320 | stereotaxic |
| Stroemer2009[62] | Temporary | rat | 86 | male | halothane | cell line | CTX0E03 cells | 4.5×10^5^ | 40320 | stereotaxic |
| Jin2010[63] | Permanent | rat | 40 | male | isoﬂurane | cell line | NPCs | 1.2×10^5^ | 30240 | stereotaxic |
| Shen2010 [64] | Temporary | rat | 160 | male | isoflurane | rat | NSCs | 1.0 ×10^7^ | 60 | systematic(IV) |
| Jiang2011 [65] | Temporary | rat | 20 | female | halothane | human | iPSCs | 8.0×10^5^ | unclear | stereotaxic |
| Tang2014 [66] | Temporary | rat | 40 | male | ketamine | rat | MSCs | 1.0×10^6^ | 1440 | stereotaxic |
| Ma2015[67] | Permanent | mouse | 29 | male | ketamine | mouse | NSCs | unclear | 2880 | systematic(IV) |
| Zhao2015 [68] | Temporary | rat | 36 | male | chloral hydrate | human | UCMSCs | unclear | 1440-20160 | intranasal |
| Gutiérrez-Fernández2011[69] | Permanent | rat | 60 | male | ketamine | rat | MSCs | 2.0 ×10^6^ | 90 | systematic(IV)  systematic(IA) |
| Otero-Ortega2015[70] | Permanent | rat | 72 | male | isoflurane | rat | ADMSCs | 2.0×10^6^ | 1440 | systematic(IV) |
| Veizovic 2001[71] | Temporary | rat | 32 | male | halothane | mouse | MHP36 cells | 7.5×10^4^ | 20160-30240 | stereotaxic |
| Kamiya2008[72] | Temporary | rat | 41 | male | halothane | rat | BMMCs | 1.0×10^7^ | 90 | systematic(IA) |
| Hicks2009 [73] | Permanent | rat | 51 | male | isoﬂurane | cell line | NPCs | 8.0×10^5^ | 10080 | stereotaxic |
| Gutiérrez-Fernández2013[74] | Permanent | rat  rat | 20  20 | male  male | ketamine ketamine | rat  rat | BMSCs ADMSCs | 2.0 ×10^6^  2.0 ×10^6^ | 90  90 | systematic(IV)  systematic(IV) |
| Huang2014 [75] | Temporary | mouse | 18 | male | isoflurane | human | NSCs | 1.0×10^5^ | 1440 | stereotaxic |
| Eckert2015 [76] | Temporary | mouse | 20 | male | isoflurane | cell line | NSCs | 1.0×10^5^ | 1440 | stereotaxic |
| Vats2019[77] | Temporary | rat | 30 | female | unclear | rat | MSCs | 1.0×10^5^ | 360 | systematic(IA) |
| Lin2017[78] | Temporary | rat | 40 | female | unclear | human | UCMSCs | 1.2×10^6^ | 1440 | systematic(IV) |
| Sibov2019[79] | Temporary | rat | 43 | female | halothane | human | AFSCs | unclear | 60 | systematic(IV) |
| Zuo2019[80] | Temporary | rat | 124 | both | pentobarbital | human | UCMSCs | 2.0×10^6^ | unclear | systematic(IV) |
| Yamashita2017[81] | Temporary | mouse | 44 | male | isoflurane | Murine | NSCs | 5.0×10^5^ | unclear | stereotaxic |
| Zhang2018[82] | Temporary | rat | 90 | male | unclear | rat | ADMSCs | 1.0×10^6^ | 11520 | stereotaxic |
| Cheng2018[83] | Temporary | mouse | 118 | male | ketamine | rat | MSCs | 1.0×10^5^ | unclear | stereotaxic |
| Abd El Motteleb2017[84] | Temporary | rat | 60 | male | unclear | human | MSCs | 1.5×10^5^ | 30 | unclear |
| Cherkashova2019[85] | Temporary | rat  rat | 30  27 | male | isoflurane  isoflurane | human  human | MSCs  NPCs | unclear  unclear | 1440  1440 | systematic(IV)  systematic(IV) |
| Park2015[86] | Temporary | rat | 48 | male | Rompun | human | MSCs | 5.0×10^5^ | 2880 | stereotaxic |
| Park2017[87] | Temporary | rat | 23 | male | xylazine | human | MSCs | 5.0×10^5^ | 2880 | stereotaxic |
| Hou2016[88] | Temporary | mouse | 79 | both | unclear | mouse | NSCs | 2.5×10^5^ | unclear | stereotaxic |
| Hosseini2015[89] | Temporary | rat  rat | 20  20 | male  male | halothane  halothane | rat  rat | MSCs  NSCs | 1.0×10^5^  1.0×10^5^ | 1440  10080 | stereotaxic |
| Chi2018[90] | Temporary | rat | 108 | male | unclear | rat | ADMSCs | 2.0×10^6^ | 0  720  1440 | systematic(IV) |
| Xie2019[91] | Temporary | rat | 70 | male | unclear | human | BMSCs | 1.0×10^7^ | 4320 | stereotaxic |
| Ryu2016[92] | Temporary | rat | 14 | male | unclear | human | NSCs | 1.2×10^5^ | 1440 | stereotaxic |
| Hosseini2020[93] | Temporary | rat | 60 | unclear | halothane | rat | NSCs | 2.0×10^5^ | -1440  5760 | stereotaxic |
| Saraf2019[94] | Temporary | rat | 30 | female | unclear | rat | MSCs | 1.0×10^5^ | unclear | systematic(IA) |
| Oh2020[95] | Temporary | rat | 29 | male | ketamine | human | iPSCs | 2.0×10^5^ | 10080 | stereotaxic |
| Vahidinia2019[96] | Temporary | rat | 28 | male | isoflurane | rat | BMSCs | 1.0×10^6^ | 180 | systematic(IV) |
| Wu2015[97] | Temporary | rat | 28 | male | chloral hydrate | human | PDMCs | 5.0×10^4^ | 0 | stereotaxic |
| Leu2010[98] | Temporary | rat | 40 | male | unclear | rat | ADMSCs | 2.0×10^6^ | 1440 | systematic(IV) |

Abbreviations: BMSCs=Bone marrow mesenchymal stem cells; MSCs=marrow stromal cells; hATSCs= human adipose tissue-derived stromal cells; UCBC=human umbilical cord blood cells; NSPCs=neural stem/progenitor cells; NSCs=neural stem cells; NPCs=neural progenitor cells; UCMSCs= umbilical cord derived mesenchymal stem cells; ADSCs= adipose-derived stem cells; ADMSCs= adipose-derived mesenchymal stem cell; iPSCs= Induced pluripotent stem cells; SP= side population; AFSCs=amniotic fluid-derived stem cells; SHED=human exfoliated deciduous tooth; BMMCs=bone marrow mononuclear cells; UTCs=umbilical tissue-derived cells; PDMCs=placenta-derived multipotent stem cells; iNSCs=induced neural stem cells; DPSCs=Dental Pulp Stem Cells; MAPCs=Multipotent Adult Progenitor Cells; EPCs=endothelial progenitor cells; IV= intravenous; IA= intra-arterial

**Table S2 Information about the passage that used for stem cells**

| Study  (years) | animal type | number of the passage | culture medium | supplement |
| --- | --- | --- | --- | --- |
| Choi2016[1] | mouse | unclear | LG-DMEM | 10%FBS |
| Jiang2018[2] | rat | 3, 4, 5 | DMEM | 10%FBS, penicillin, streptomycin |
| Zhao2020[3] | rat | 3 | LG-DMEM | 10%FBS |
| Tian2019[4] | rat | unclear | DMEM | 10%FBS, FGF, EGF, penicillin, streptomycin |
| Zhang2011 [5] | rat | unclear | non hematopoietic stem cell medium | penicillin, streptomycin |
| Inoue2013 [6] | rat | 3, 4, 5 | DMEM | antibiotic, mesenchymal cell growth supplement |
| Du2014[7] | rat | 3 | LG-DMEM | 10%FBS, antibiotic |
| Liu2009[8] | rat | 3 | DMEM | 10%FBS, antibiotic |
| Tobin2020[9] | rat | unclear | unclear | unclear |
| Zong2017[10] | rat | unclear | LG-DMEM | 10%FBS |
| He2018[11] | rat | 3 | DMEM | 15%FBS |
| Nito2018[12] | rat | 3 | DMEM | 10%FBS |
| Zhang2017[13] | rat | unclear | DMEM | 10%FBS, 5% horse serum, penicillin, streptomycin |
| Souza2017[14] | rat | unclear | unclear | unclear |
| Li2015[15] | rat | unclear | DMEM | 15%FBS |
| Son2019[16] | rat | 6, 7, 8, 9, 10, 11, 12 | LG-DMEM | 10%FBS |
| Huang2018[17] | rat | unclear | unclear | unclear |
| Zhang2018[18] | rat | 2 | DMEM | EGF, FGF, penicillin, streptomycin |
| Yang2015[19] | rat | unclear | LG-DMEM | 10%FBS |
| Liu2011[20] | rat | 3 | DMEM | 10%FBS, penicillin, streptomycin, amphotericin B |
| Zhang2006 [21] | rat | 3, 4 | α-MEM | 10%FBS, penicillin, streptomycin, amphotericin B |
| Shen2007 [22] | rat | unclear | unclear | unclear |
| Koh2008 [23] | rat | unclear | unclear | unclear |
| Lim2011[24] | rat | 5, 6, 7, 8 | α-MEM | 10%FBS |
| Bao2011[25] | rat | 5 | DMEM | 10%FBS, penicillin, EGF, PDGF |
| Ikegame2011[26] | mouse | 2 | DMEM | 10%FBS, 1% antibiotic |
| Song2011 [27] | rat | unclear | DMEM | 5%FBS, 5% horse serum, penicillin, streptomycin, EGF, FGF |
| sugiyama2011[28] | rat | unclear | EBM-2 | 10%FBS, IGF1, EGF, VEGF |
| Wang2013 [29] | rat | 1 | DMEM | 1%FBS, GF |
| Jensen2013 [30] | rat | unclear | ESM | FGF-2 |
| Cheng2015 [31] | rat | unclear | DMEM | 10%FBS, 5% horse serum, penicillin, streptomycin |
| Boltze2006 [32] | rat | unclear | unclear | unclear |
| Cho2010[33] | rat | unclear | HG-DMEM, MEM | 10%FBS |
| Mitkari2014 [34] | rat | 3 | LG-DMEM | plasma, penicillin, streptomycin |
| Lam2020[35] | rat | unclear | DMEM | 10%FBS, penicillin, streptomycin |
| Yamaguchi2018[36] | rat | unclear | normal media | FGF |
| Gutiérrez-Fernández2015[37] | rat | 3 | DMEM | 20%FBS, penicillin, streptomycin |
| Zhang2017[38] | mouse | 3, 4, 5 | DMEM | FGF, EGF |
| Lin2020[39] | rat | unclear | DMEM | 10%FBS |
| Salehi2020[40] | rat | 4, 5, 6 | α-MEM | 10%FBS, penicillin, streptomycin |
| Kong2018[41] | rat | 5 | α-MEM | 10%FBS, penicillin, streptomycin |
| Yang2018[42] | rat | unclear | DMEM | 10%FBS |
| Zhu2017[43] | rat | 3 | DMEM | 4%FBS |
| Bacigaluppi2016[44] | mouse | 15 | unclear | unclear |
| Li2018[45] | rat | 3, 4 | LG-DMEM | 11%FBS, penicillin, streptomycin |
| Sowa2018[46] | rat | 7, 8, 9 | DMEM | 10%FBS, antibiotic |
| Paudyal2020[47] | rat | unclear | DMEM | 10%FBS |
| Bi2018[48] | rat | 3 | DMEM | 15%FBS, penicillin G, streptomycin |
| Ryu2018[49] | rat | 5, 6 | DMEM | 10%FBS, penicillin, streptomycin |
| Wang2015[50] | mouse | unclear | α-MEM | 5%FBS, gentamycin |
| Yang2017[51] | rat | unclear | unclear | unclear |
| Zhang2018[52] | rat | 2 | DMEM | 10%FBS, penicillin, streptomycin |
| Moisan2016[53] | human | 2 | unclear | unclear |
| Zhao2002[54] | rat | 26 | DMEM | dexamethasone, penicillin, streptomycin |
| Kurozumi2004[55] | rat | 40 | DMEM | 10%FBS |
| Li2005[56] | rat | unclear | unclear | unclear |
| Wu2008[57] | rat | unclear | IMDM | 10%FBS, penicillin G, streptomycin |
| Andrews2008[58] | rat | unclear | unclear | unclear |
| Li2008[59] | rat | 6 | DMEM | 10%FBS |
| Liao2009 [60] | rat | 4, 5, 6 | α-MEM | 10%FBS, penicillin, streptomycin, EGF |
| Chen2009 [61] | rat | 4 | DMEM | proliferation: 4%FBS |
| Stroemer2009[62] | rat | 1 | normal media | growth factors: FGF, EGF |
| Jin2010[63] | rat | unclear | DMEM | LIF, FGF-2 |
| Shen2010 [64] | rat | 1 | DMEM | insulin |
| Jiang2011 [65] | rat | unclear | standard human embryonic stem cell culture medium | unclear |
| Tang2014 [66] | rat | 3, 4, 5, 6 | HG-DMEM | 10%FBS |
| Ma2015[67] | mouse | 5, 6 | DMEM | 10%FBS, antibiotic, EGF |
| Zhao2015 [68] | rat | 3, 4, 5 | DMEM | 10%FBS |
| Gutiérrez-Fernández2011[69] | rat | 3 | DMEM | 20%FBS, penicillin, streptomycin |
| Otero-Ortega2015[70] | rat | 3 | unclear | unclear |
| Veizovic 2001[71] | rat | unclear | unclear | unclear |
| Kamiya2008[72] | rat | 1 | unclear | unclear |
| Hicks2009 [73] | rat | 44 | DMEM | 20%FBS, penicillin, streptomycin, FGF |
| Gutiérrez-Fernández2013[74] | rat | 3 | DMEM | 20%FBS, penicillin, streptomycin |
| Huang2014 [75] | mouse | unclear | HG-DMEM | FGF, EGF, LIF |
| Eckert2015 [76] | mouse | 3 | DMEM | EGF, FGF |
| Vats2019[77] | rat | 5 | DMEM | 10%FBS |
| Lin2017[78] | rat | unclear | normal media | unclear |
| Sibov2019[79] | rat | 3 | α-MEM,  LG-DMEM | α-MEM: 15%FBS  LG-DMEM: penicillin, streptomycin, amphotericin B |
| Zuo2019[80] | rat | unclear | LG-DMEM | 10%FBS, penicillin, streptomycin |
| Yamashita2017[81] | mouse | unclear | DMEM | EGF, FGF, penicillin, streptomycin |
| Zhang2018[82] | rat | 3 | DMEM | unclear |
| Cheng2018[83] | mouse | unclear | DMEM | 10%FBS |
| Abd El Motteleb2017[84] | rat | unclear | DMEM | 10%FBS |
| Cherkashova2019[85] | rat | 5 | DMEM | 10%FBS, penicillin, streptomycin |
| Park2015[86] | rat | unclear | α-MEM | 10%FBS |
| Park2017[87] | rat | unclear | α-MEM | 10%FBS |
| Hou2016[88] | mouse | 3 | DMEM | EGF, FGF |
| Hosseini2015[89] | rat | unclear | DMEM | EGF, FGF |
| Chi2018[90] | rat | unclear | DMEM | unclear |
| Xie2019[91] | rat | 5 | DMEM | 10%FBS |
| Ryu2016[92] | rat | unclear | DMEM | 5%FBS, 5% horse serum, penicillin,  streptomycin, EGF, FGF |
| Hosseini2020[93] | rat | unclear | DMEM | 5%FBS, 10%FBS, EGF, FGF |
| Saraf2019[94] | rat | unclear | unclear | unclear |
| Oh2020[95] | rat | unclear | DMEM | 5% knock-out serum replacement, FGF |
| Vahidinia2019[96] | rat | 3, 4, 5 | DMEM  LG-DMEM | 10%FBS, penicillin, streptomycin |
| Wu2015[97] | rat | unclear | unclear | unclear |
| Leu2010[98] | rat | unclear | DMEM | 10%FBS |

Abbreviations: DMEM= dulbecco's modified eagle medium; α-MEM= a-minimum essential medium; IMDM= iscove's modified dulbecco medium; ESM= embryonic stem cell medium; LG-DMEM= low-glucose dulbecco's modified eagle's medium; HG-DMEM= high-glucose dulbecco's modified eagle's medium; EBM-2= endothelial basal medium-2; FBS= fetal bovine serum; EGF= epidermal growth factor; FGF= fibroblast growth factor; LIF= leukemia inhibitory factor; PDGF= platelet-derived growth factor; IGF1= insulin-like growth factor 1; VEGF= vascular endothelial growth factor.

**Table S3 Quality assessment of the included studies (References is on the next page)**

|  | (1)  Peer reviewed publication | (2)  Control of temperature | (3)  Random allocation | (4)  Blinded assessment | (5)  Anesthetic without neuroprotective activity | (6)  Sample size calculation | (7)  Appropriate animal model | (8)  Compliance with animal welfare regulations | (9)  Statement of conflict of interests | Quality score |
| --- | --- | --- | --- | --- | --- | --- | --- | --- | --- | --- |
| Choi2016[1] | √ | √ | √ | √ | √ | ? | √ | √ | √ | 8 |
| Jiang2018[2] | √ | √ | √ | √ | √ | ? | √ | √ | √ | 8 |
| Zhao2020[3] | √ | √ | √ | √ | √ | ? | √ | √ | √ | 8 |
| Tian2019[4] | √ | √ | √ | √ | √ | ? | √ | √ | √ | 8 |
| Zhang2011 [5] | √ | ? | √ | √ | √ | ? | √ | √ | √ | 7 |
| Inoue2013 [6] | √ | √ | √ | √ | ? | ? | √ | √ | √ | 7 |
| Du2014[7] | √ | √ | √ | √ | √ | ? | √ | √ | ? | 7 |
| Liu2009[8] | √ | √ | √ | √ | √ | ? | √ | √ | ? | 7 |
| Tobin2020[9] | √ | √ | √ | √ | ? | √ | √ | √ | ? | 7 |
| Zong2017[10] | √ | √ | √ | √ | √ | ? | √ | √ | ? | 7 |
| He2018[11] | √ | √ | √ | ? | √ | ? | √ | √ | √ | 7 |
| Nito2018[12] | √ | √ | √ | √ | ? | ? | √ | √ | √ | 7 |
| Zhang2017[13] | √ | √ | ? | √ | √ | ? | √ | √ | √ | 7 |
| Souza2017[14] | √ | √ | √ | √ | ? | ? | √ | √ | √ | 7 |
| Li2015[15] | √ | √ | √ | √ | √ | ? | √ | ? | √ | 7 |
| Son2019[16] | √ | √ | √ | √ | √ | ? | √ | √ | ? | 7 |
| Huang2018[17] | √ | √ | √ | √ | ? | ? | √ | √ | √ | 7 |
| Zhang2018[18] | √ | √ | √ | √ | √ | ? | √ | √ | ? | 7 |
| Yang2015[19] | √ | √ | √ | √ | ? | ? | √ | √ | √ | 7 |
| Liu2011[20] | √ | √ | √ | ? | √ | ? | √ | √ | √ | 7 |
| Zhang2006 [21] | √ | √ | √ | √ | ? | ? | √ | √ | ? | 6 |
| Shen2007 [22] | √ | √ | √ | √ | ? | ? | √ | √ | ? | 6 |
| Koh2008 [23] | √ | √ | √ | √ | ? | ? | √ | √ | ? | 6 |
| Lim2011[24] | √ | √ | √ | ? | ? | ? | √ | √ | √ | 6 |
| Bao2011[25] | √ | √ | √ | ? | √ | ? | √ | √ | ? | 6 |
| Ikegame2011[26] | √ | √ | ? | √ | ? | ? | √ | √ | √ | 6 |
| Song2011 [27] | √ | √ | ? | √ | ? | ? | √ | √ | √ | 6 |
| sugiyama2011[28] | √ | √ | √ | ? | ? | ? | √ | √ | √ | 6 |
| Wang2013 [29] | √ | √ | √ | √ | ? | ? | √ | √ | ? | 6 |
| Jensen2013 [30] | √ | √ | √ | √ | ? | ? | √ | √ | ? | 6 |
| Cheng2015 [31] | √ | √ | √ | ? | ? | ? | √ | √ | √ | 6 |
| Boltze2006 [32] | √ | √ | √ | √ | ? | ? | √ | √ | ? | 6 |
| Cho2010[33] | √ | √ | √ | √ | ? | ? | √ | √ | ? | 6 |
| Mitkari2014 [34] | √ | √ | √ | √ | ? | ? | √ | √ | ? | 6 |
| Lam2020[35] | √ | √ | √ | ? | ? | ? | √ | √ | √ | 6 |
| Yamaguchi2018[36] | √ | ? | √ | √ | ? | ? | √ | √ | √ | 6 |
| Gutiérrez-Fernández2015[37] | √ | ? | √ | √ | ? | ? | √ | √ | √ | 6 |
| Zhang2017[38] | √ | √ | √ | √ | ? | ? | √ | ? | √ | 6 |
| Lin2020[39] | √ | √ | √ | √ | ? | ? | √ | ? | √ | 6 |
| Salehi2020[40] | √ | √ | √ | ? | ? | ? | √ | √ | √ | 6 |
| Kong2017[41] | √ | ? | √ | √ | ? | ? | √ | √ | √ | 6 |
| Yang2018[42] | √ | ? | √ | √ | ? | ? | √ | √ | √ | 6 |
| Zhu2017[43] | √ | √ | √ | √ | ? | ? | √ | √ | ? | 6 |
| Bacigaluppi2016[44] | √ | √ | √ | √ | ? | ? | √ | √ | ? | 6 |
| Li2018[45] | √ | √ | √ | ? | ? | ? | √ | √ | √ | 6 |
| Sowa2018[46] | √ | √ | ? | √ | ? | ? | √ | √ | √ | 6 |
| Paudyal2020[47] | √ | ? | √ | √ | ? | ? | √ | √ | √ | 6 |
| Bi2018[48] | √ | √ | √ | √ | ? | ? | √ | √ | ? | 6 |
| Ryu2018[49] | √ | √ | √ | √ | ? | ? | √ | √ | ? | 6 |
| Wang2015[50] | √ | √ | √ | √ | ? | ? | √ | √ | ? | 6 |
| Yang2017[51] | √ | √ | ? | √ | ? | ? | √ | √ | √ | 6 |
| Zhang2018[52] | √ | ? | ? | √ | √ | ? | √ | √ | √ | 6 |
| Moisan2016[53] | √ | √ | √ | ? | ? | ? | √ | √ | √ | 6 |
| Zhao2002[54] | √ | √ | √ | √ | ? | ? | √ | ? | ? | 5 |
| Kurozumi2004[55] | √ | √ | √ | ? | ? | ? | √ | √ | ? | 5 |
| Li2005[56] | √ | √ | √ | √ | ? | ? | √ | ? | ? | 5 |
| Wu2008[57] | √ | ? | √ | √ | ? | ? | √ | √ | ? | 5 |
| Andrews2008[58] | √ | √ | √ | ? | ? | ? | √ | √ | ? | 5 |
| Li2008[59] | √ | √ | √ | √ | ? | ? | √ | ? | ? | 5 |
| Liao2009 [60] | √ | √ | ? | √ | √ | ? | √ | ? | ? | 5 |
| Chen2009 [61] | √ | √ | ? | √ | ? | ? | √ | √ | ? | 5 |
| Stroemer2009[62] | √ | √ | √ | ? | ? | ? | √ | √ | ? | 5 |
| Jin2010[63] | √ | √ | ? | √ | ? | ? | √ | √ | ? | 5 |
| Shen2010 [64] | √ | √ | √ | ? | ? | ? | √ | √ | ? | 5 |
| Jiang2011 [65] | √ | √ | ? | √ | ? | ? | √ | ? | √ | 5 |
| Tang2014 [66] | √ | √ | √ | √ | ? | ? | √ | ? | ? | 5 |
| Ma2015[67] | √ | √ | ? | ? | ? | ? | √ | √ | √ | 5 |
| Zhao2015 [68] | √ | √ | √ | ? | ? | ? | √ | √ | ? | 5 |
| Gutiérrez-Fernández2011[69] | √ | ? | √ | √ | ? | ? | √ | √ | ? | 5 |
| Otero-Ortega2015[70] | √ | √ | ? | ? | ? | ? | √ | √ | √ | 5 |
| Veizovic 2001[71] | √ | √ | √ | ? | ? | ? | √ | √ | ? | 5 |
| Kamiya2008[72] | √ | √ | ? | √ | ? | ? | √ | √ | ? | 5 |
| Hicks2009 [73] | √ | √ | √ | ? | ? | ? | √ | √ | ? | 5 |
| Gutiérrez-Fernández2013[74] | √ | ? | √ | ? | ? | ? | √ | √ | √ | 5 |
| Huang2015 [75] | √ | √ | ? | ? | ? | ? | √ | √ | √ | 5 |
| Eckert2015 [76] | √ | √ | ? | ? | ? | ? | √ | √ | √ | 5 |
| Vats2019[77] | √ | √ | ? | ? | ? | ? | √ | √ | √ | 5 |
| Lin2017[78] | √ | ? | √ | ? | ? | ? | √ | √ | √ | 5 |
| Sibov2019[79] | √ | √ | ? | ? | ? | ? | √ | √ | √ | 5 |
| Zuo2019[80] | √ | ? | √ | ? | ? | ? | √ | √ | √ | 5 |
| Yamashita2017[81] | √ | √ | ? | ? | ? | ? | √ | √ | √ | 5 |
| Zhang2018[82] | √ | ? | √ | √ | ? | ? | √ | ? | √ | 5 |
| Cheng2018[83] | √ | ? | ? | √ | ? | ? | √ | √ | √ | 5 |
| Abd El Motteleb2017[84] | √ | ? | √ | ? | ? | ? | √ | √ | √ | 5 |
| Cherkashova2019[85] | √ | √ | √ | ? | ? | ? | √ | √ | ? | 5 |
| Park2015[86] | √ | √ | ? | ? | √ | ? | √ | √ | ? | 5 |
| Park2017[87] | √ | ? | √ | ? | √ | ? | √ | √ | ? | 5 |
| Hou2016[88] | √ | √ | √ | ? | ? | ? | √ | ? | √ | 5 |
| Hosseini2015[89] | √ | ? | √ | ? | ? | ? | √ | √ | √ | 5 |
| Chi2018[90] | √ | ? | √ | √ | ? | ? | √ | √ | ? | 5 |
| Xie2019[91] | √ | ? | √ | ? | ? | ? | √ | √ | √ | 5 |
| Ryu2016[92] | √ | √ | ? | ? | ? | ? | √ | √ | √ | 5 |
| Hosseini2020[93] | √ | ? | √ | ? | ? | ? | √ | √ | √ | 5 |
| Saraf2019[94] | √ | √ | ? | ? | ? | ? | √ | √ | √ | 5 |
| Oh2020[95] | √ | √ | ? | ? | ? | ? | √ | √ | √ | 5 |
| Vahidinia2019[96] | √ | √ | √ | √ | ? | ? | √ | ? | ? | 5 |
| Wu2015[97] | √ | √ | ? | ? | √ | ? | √ | √ | ? | 5 |
| Leu2010[98] | √ | ? | ? | √ | ? | ? | √ | √ | √ | 5 |

1. Choi YK, Urnukhsaikhan E, Yoon HH, Seo YK and Park JK. Effect of human mesenchymal stem cell transplantation on cerebral ischemic volume-controlled photothrombotic mouse model. Biotechnol J 2016;1111(11).
2. Jiang RH, Wu CJ, Xu XQ et al. Hypoxic conditioned medium derived from bone marrow mesenchymal stromal cells protects against ischemic stroke in rats. J. Cell. Physiol. 2019;2342(2).
3. Zhao K, Li R, Bi S et al. Combination of mild therapeutic hypothermia and adipose-derived stem cells for ischemic brain injury. Neural Regen Res 2018;1310(10).
4. Tian L, Zhu W, Liu Y et al. Neural Stem Cells Transfected with Leukemia Inhibitory Factor Promote Neuroprotection in a Rat Model of Cerebral Ischemia. Neurosci Bull 2019;355(5).
5. Zhang Z, Jin D, Yang Z et al. Effects of 17 beta-estradiol pre-treated adult neural stem cells on neuronal differentiation and neurological recovery in rats with cerebral ischemia. Brain Injury 2011;25:227-236.
6. Inoue T, Sugiyama M, Hattori H et al. Stem cells from human exfoliated deciduous tooth-derived conditioned medium enhance recovery of focal cerebral ischemia in rats. Tissue eng Part A 2013;19:24-29.
7. Du G, Liu Y, Dang M et al. Comparison of administration routes for adipose-derived stem cells in the treatment of middle cerebral artery occlusion in rats. Acta Histochemica 2014;116:1075-1084.
8. Liu N, Chen R, Du H et al. Expression of IL-10 and TNF-alpha in rats with cerebral infarction after transplantation with mesenchymal stem cells. Cell Mol Immunol 2009;6:207-213.
9. Tobin MK, Stephen TKL, Lopez KL et al. Activated Mesenchymal Stem Cells Induce Recovery Following Stroke Via Regulation of Inflammation and Oligodendrogenesis. J Am Heart Assoc 2020;97(7).
10. Zong X, Wu S, Li F et al. Transplantation of VEGF-mediated bone marrow mesenchymal stem cells promotes functional improvement in a rat acute cerebral infarction model. Brain Res. 2017;1676.
11. He H, Zeng Q, Huang G et al. Bone marrow mesenchymal stem cell transplantation exerts neuroprotective effects following cerebral ischemia/reperfusion injury by inhibiting autophagy via the PI3K/Akt pathway. Brain Res. 2019;1707.
12. Nito C, Sowa K, Nakajima M et al. Transplantation of human dental pulp stem cells ameliorates brain damage following acute cerebral ischemia. Biomed. Pharmacother. 2018;108.
13. Zhang JJ, Zhu JJ, Hu YB et al. Transplantation of bFGF-expressing neural stem cells promotes cell migration and functional recovery in rat brain after transient ischemic stroke. Oncotarget, 2017;8(60).
14. Souza CC, da Silva MC, Lopes RT et al. Comparative Therapeutic Effects of Minocycline Treatment and Bone Marrow Mononuclear Cell Transplantation following Striatal Stroke. Oxid Med Cell Longev 2017;2017.
15. Li Y, Mao WW, Zhang CG et al. Neuroprotective effects of intravenous transplantation of bone marrow mononuclear cells from 5-fluorouracil pre-treated rats on ischemic stroke. Behav. Brain Res. 2016;301.
16. Son JW, Park J, Kim YE et al. Glia-Like Cells from Late-Passage Human MSCs Protect Against Ischemic Stroke Through IGFBP-4. Mol. Neurobiol. 2019;5611(11).
17. Huang Y, Wang J, Cai J et al. Targeted homing of CCR2-overexpressing mesenchymal stromal cells to ischemic brain enhances post-stroke recovery partially through PRDX4-mediated blood-brain barrier preservation. Theranostics 2018;821(21).
18. Zhang HL, Xie XF, Xiong YQ et al. Comparisons of the therapeutic effects of three different routes of bone marrow mesenchymal stem cell transplantation in cerebral ischemic rats. Brain Res. 2018;1680.
19. Yang Z, Cai X, Xu A et al. Bone marrow stromal cell transplantation through tail vein injection promotes angiogenesis and vascular endothelial growth factor expression in cerebral infarct area in rats. Cytotherapy 2015;179(9).
20. Liu N, Zhang Y, Fan L et al. Effects of transplantation with bone marrow-derived mesenchymal stem cells modified by Survivin on experimental stroke in rats. J Transl Med 2011 06;9.
21. Zhang C, Li Y, Chen J et al. Bone marrow stromal cells upregulate expression of bone morphogenetic proteins 2 and 4, gap junction protein connexin-43 and synaptophysin after stroke in rats. Neuroscience 2006;141:687-695.
22. Shen LH, Li Y, Chen J et al. Therapeutic benefit of bone marrow stromal cells administered 1 month after stroke. J Cereb Blood Flow Metab 2007;27:6-13.
23. Koh S-H, Kim KS, Choi MR et al. Implantation of human umbilical cord-derived mesenchymal stem cells as a neuroprotective therapy for ischemic stroke in rats. Brain Research 2008;1229:233-248.
24. Lim J, Jeong C, Jun J et al. Therapeutic effects of human umbilical cord blood-derived mesenchymal stem cells after intrathecal administration by lumbar puncture in a rat model of cerebral ischemia. Stem Cell Research and Therapy 2011;2:38.
25. Bao X, Wei J, Feng M et al. Transplantation of human bone marrow-derived mesenchymal stem cells promotes behavioral recovery and endogenous neurogenesis after cerebral ischemia in rats. Brain Research 2011;1367:103-113.
26. Ikegame Y, Yamashita K, Hayashi S-I et al. Comparison of mesenchymal stem cells from adipose tissue and bone marrow for ischemic stroke therapy. Cytotherapy 2011;13:675-685.
27. Song M, Kim Y-J, Kim Y-H et al. Effects of Duplicate Administration of Human Neural Stem Cell After Focal Cerebral Ischemia in the Rat. International Journal of Neuroscience 2011;121:457-461.
28. Sugiyama M, Iohara K, Wakita H et al. Dental pulp-derived CD31/CD146 side population stem/progenitor cells enhance recovery of focal cerebral ischemia in rats. Tissue eng Part A 2011;17:1303-1311.
29. Wang H, Nagai A, Sheikh AM et al. Human mesenchymal stem cell transplantation changes proinflammatory gene expression through a nuclear factor-kappaB-dependent pathway in a rat focal cerebral ischemic model. J Neurosci Res 2013;91:1440-1449.
30. Jensen MB, Yan H, Krishnaney-Davison R et al. Survival and differentiation of transplanted neural stem cells derived from human induced pluripotent stem cells in a rat stroke model. J Stroke Cerebrovasc Dis 2013;22:304-308.
31. Cheng Y, Zhang J, Deng L et al. Intravenously delivered neural stem cells migrate into ischemic brain, differentiate and improve functional recovery after transient ischemic stroke in adult rats. Int J Clin Exp Pathol 2015;8:2928-2936.
32. Boltze J, Kowalski I, Förschler A et al. The stairway: a novel behavioral testdetecting sensomotoric stroke deficits in rats. Artif Organs 2006;30:756-63.
33. Cho GW, Koh SH, Kim MH et al. The neuroprotective effect of erythropoietin-transduced human mesenchymal stromal cells in an animal model of ischemic stroke. Brain Research 2010;1353:1-13.
34. Lapi D, Vagnani S, Sapio D et al. Effects of bone marrow mesenchymal stem cells (BM-MSCs) on rat pial microvascular remodeling after transient middle cerebral artery occlusion. Frontiers in Cellular Neuroscience 2015; 9:329.
35. Lam PK, Wang KKW, Chin DWC et al. Topically applied adipose-derived mesenchymal stem cell treatment in experimental focal cerebral ischemia. J Clin Neurosci 2020 ;71.
36. Yamaguchi S, Horie N, Satoh K et al. Age of donor of human mesenchymal stem cells affects structural and functional recovery after cell therapy following ischaemic stroke. J. Cereb. Blood Flow Metab. 2018;387(7).
37. Gutiérrez-Fernández M, Rodríguez-Frutos B, Ramos-Cejudo J et al. Comparison between xenogeneic and allogeneic adipose mesenchymal stem cells in the treatment of acute cerebral infarct: proof of concept in rats. J Transl Med 2015;13.
38. Zhang T, Yang X, Liu T et al. Adjudin-preconditioned neural stem cells enhance neuroprotection after ischemia reperfusion in mice. Stem Cell Res Ther 2017;81(1).
39. Lin KC, Chai HT, Chen KH et al. Intra-carotid arterial transfusion of circulatory-derived autologous endothelial progenitor cells in rodent after ischemic stroke-evaluating the impact of therapeutic time points on prognostic outcomes. Stem Cell Res Ther 2020;111(1).
40. Salehi MS, Pandamooz S, Safari A et al. Epidermal neural crest stem cell transplantation as a promising therapeutic strategy for ischemic stroke. CNS Neurosci Ther 2020;267(7).
41. Kong T, Park JM, Jang JH et al. Immunomodulatory effect of CD200-positive human placenta-derived stem cells in the early phase of stroke. Exp. Mol. Med. 2018;501(1).
42. Yang B, Li W, Satani N et al. Protective Effects of Autologous Bone Marrow Mononuclear Cells After Administering t-PA in an Embolic Stroke Model. Transl Stroke Res 2018;92(2).
43. Zhu JD, Wang JJ, Ge G and Kang CS. Effects of Noggin-Transfected Neural Stem Cells on Neural Functional Recovery and Underlying Mechanism in Rats with Cerebral Ischemia Reperfusion Injury. J Stroke Cerebrovasc Dis 2017;267(7).
44. Bacigaluppi M, Russo GL, Peruzzotti-Jametti L et al. Neural Stem Cell Transplantation Induces Stroke Recovery by Upregulating Glutamate Transporter GLT-1 in Astrocytes. J. Neurosci. 2016;3641(41).
45. Li X, Huang M, Zhao R et al. Intravenously Delivered Allogeneic Mesenchymal Stem Cells Bidirectionally Regulate Inflammation and Induce Neurotrophic Effects in Distal Middle Cerebral Artery Occlusion Rats Within the First 7 Days After Stroke. Cell. Physiol. Biochem. 2018;465(5).
46. Sowa K, Nito C, Nakajima M et al. Impact of Dental Pulp Stem Cells Overexpressing Hepatocyte Growth Factor after Cerebral Ischemia/Reperfusion in Rats. Mol Ther Methods Clin Dev 2018;10.
47. Paudyal A, Ghinea FS, Driga MP et al. p5 Peptide-Loaded Human Adipose-Derived Mesenchymal Stem Cells Promote Neurological Recovery After Focal Cerebral Ischemia in a Rat Model. Transl Stroke Res 2020.
48. Bi M, Wang J, Zhang Y et al. Bone mesenchymal stem cells transplantation combined with mild hypothermia improves the prognosis of cerebral ischemia in rats. PLoS ONE 2018;138(8).
49. Ryu B, Sekine H, Homma J et al. Allogeneic adipose-derived mesenchymal stem cell sheet that produces neurological improvement with angiogenesis and neurogenesis in a rat stroke model. J. Neurosurg. 2019;1322(2).
50. Wang J, Xia J, Zhang F et al. Galectin-1-secreting neural stem cells elicit long-term neuroprotection against ischemic brain injury. Sci Rep 2015;5.
51. Yang B, Hamilton JA, Valenzuela KS et al. Multipotent Adult Progenitor Cells Enhance Recovery After Stroke by Modulating the Immune Response from the Spleen. Stem Cells 2017;355(5).
52. Zhang G, Chen L, Chen W et al. γNeural Stem Cells Alleviate Inflammation via Neutralization of IFN- Negative Effect in Ischemic Stroke Model. J Biomed Nanotechnol 2018;146(6).
53. Moisan A, Favre I, Rome C et al. Intravenous Injection of Clinical Grade Human MSCs After Experimental Stroke: Functional Benefit and Microvascular Effect. Cell Transplant 2016;2512(12).
54. Zhao LR, Duan WM, Reyes M et al. Human bone marrow stem cells exhibit neural phenotypes and ameliorate neurological deficits after grafting into the ischemic brain of rats. Experimental Neurology 2002;174:11-20.
55. Kurozumi K, Nakamura K, Tamiya T et al. BDNF gene-modified mesenchymal stem cells promote functional recovery and reduce infarct size in the rat middle cerebral artery occlusion model. Molecular Therapy 2004;9:189-197.
56. Li Y, Chen J, Zhang CL et al. Gliosis and brain remodeling after treatment of stroke in rats with marrow stromal cells. Glia 2005;49:407-417.
57. Wu J, Sun Z, Sun HS et al. Intravenously administered bone marrow cells migrate to damaged brain tissue and improve neural function in ischemic rats. Cell Transplant 2008;16:993-1005.
58. Andrews EM, Tsai SY, Johnson SC et al. Human adult bone marrow-derived somatic cell therapy results in functional recovery and axonal plasticity following stroke in the rat. Experimental Neurology 2008;211:588-592.
59. Li WY, Choi YJ, Lee PH et al. Mesenchymal stem cells for ischemic stroke: changes in effects after ex vivo culturing. Cell Transplant 2008;17:1045-1059.
60. Liao W, Xie J, Zhong J et al. Therapeutic effect of human umbilical cord multipotent mesenchymal stromal cells in a rat model of stroke. Transplantation 2009;87:350-359.
61. Chen B, Gao XQ, Yang CX et al. Neuroprotective effect of grafting GDNF gene-modified neural stem cells on cerebral ischemia in rats. Brain Research 2009;1284:1-11.
62. Stroemer P, Patel S, Hope A et al. The neural stem cell line CTX0E03 promotes behavioral recovery and endogenous neurogenesis after experimental stroke in a dose-dependent fashion. Neurorehabil Neural Repair 2009;23:895-909.
63. Jin K, Mao X, Xie L et al. Delayed transplantation of human neural precursor cells improves outcome from focal cerebral ischemia in aged rats. Aging Cell 2010;9:1076-1083.
64. Shen C-C, Lin C-H, Yang Y-C et al. Intravenous Implanted Neural Stem Cells Migrate to Injury Site, Reduce Infarct Volume, and Improve Behavior after Cerebral Ischemia. Current Neurovascular Research 2010;7:167-179.
65. Jiang M, Lv L, Ji H, Yang X, Zhu W, Cai L, Gu X, Chai C, Huang S, Sun J and Dong Q. Induction of pluripotent stem cells transplantation therapy for ischemic stroke. Mol Cell Biochem 2011;354:67-75.
66. Tang Y, Cai B, Yuan F et al. Melatonin pretreatment improves the survival and function of transplanted mesenchymal stem cells after focal cerebral ischemia. Cell Transplantation 2014;23:1279-1291.
67. Ma J, Gao J, Hou B et al. Neural stem cell transplantation promotes behavioral recovery in a photothrombosis stroke model. Int J Clin Exp Pathol 2015;8:7838-7848.
68. Zhao Q, Hu J, Xiang J et al. Intranasal administration of human umbilical cord mesenchymal stem cells-conditioned medium enhances vascular remodeling after stroke. Brain Res 2015;1624:489-496.
69. Gutiérrez-Fernández M, Rodríguez-Frutos B, Alvarez-Grech J et al. Functional recovery after hematic administration of allogenic mesenchymal stem cells in acute ischemic stroke in rats. Neuroscience 2011;175:394-405.
70. Otero-Ortega L, Gutierrez-Fernandez M, Ramos-Cejudo J et al. White matter injury restoration after stem cell administration in subcortical ischemic stroke. Stem Cell Research & Therapy 2015; 6:121.
71. Veizovic T, Beech JS, Stroemer RP et al. Resolution of stroke deficits following contralateral grafts of conditionally immortal neuroepithelial stem cells. Stroke 2001;32:1012-1019.
72. Kamiya N, Ueda M, Igarashi H et al. Intra-arterial transplantation of bone marrow mononuclear cells immediately after reperfusion decreases brain injury after focal ischemia in rats. Life Sci 2008;83:433-437.
73. Hicks AU, Lappalainen RS, Narkilahti S et al. Transplantation of human embryonic stem cell-derived neural precursor cells and enriched environment after cortical stroke in rats: cell survival and functional recovery. Eur J Neurosci 2009;29:562-574.
74. Gutiérrez-Fernández M, Rodríguez-Frutos B, Ramos-Cejudo J et al. Effects of intravenous administration of allogenic bone marrow- and adipose tissue-derived mesenchymal stem cells on functional recovery and brain repair markers in experimental ischemic stroke. Stem Cell Research and Therapy 2013; 4:11.
75. Huang L, Wong S, Snyder EY et al. Human neural stem cells rapidly ameliorate symptomatic inflammation in early-stage ischemic-reperfusion cerebral injury. Stem Cell Research and Therapy 2014;5:129.
76. Eckert A, Huang L, Gonzalez R et al. Bystander Effect Fuels Human Induced Pluripotent Stem Cell-Derived Neural Stem Cells to Quickly Attenuate Early Stage Neurological Deficits After Stroke. Stem Cells Transl Med 2015;4:841-851.
77. Vats K, Sarmah D, Datta A et al. Intra-arterial Stem Cell Therapy Diminishes Inflammasome Activation After Ischemic Stroke: a Possible Role of Acid Sensing Ion Channel 1a. J. Mol. Neurosci. 2019.
78. Lin W, Hsuan YC, Lin MT et al. Human Umbilical Cord Mesenchymal Stem Cells Preserve Adult Newborn Neurons and Reduce Neurological Injury after Cerebral Ischemia by Reducing the Number of Hypertrophic Microglia/Macrophages. Cell Transplant 2017;2611(11).
79. Sibov TT, Pavon LF, Cabral FR et al. Intravenous Grafts of Human Amniotic Fluid-Derived Stem Cells Reduce Behavioral Deficits in Experimental Ischemic Stroke. Cell Transplant 2019;28.
80. Zuo L, Feng Q, Han Y et al. Therapeutic effect on experimental acute cerebral infarction is enhanced after nanoceria labeling of human umbilical cord mesenchymal stem cells. Ther Adv Neurol Disord 2019;12.
81. Yamashita T, Liu W, Matsumura Y et al. Novel Therapeutic Transplantation of Induced Neural Stem Cells for Stroke. Cell Transplant 2017;263(3).
82. Yu Z, Wenyan T, Xuewen S et al. Immunological effects of the intraparenchymal administration of allogeneic and autologous adipose-derived mesenchymal stem cells after the acute phase of middle cerebral artery occlusion in rats. J Transl Med 2018;161(1).
83. Cheng Z, Wang L, Qu M et al. Mesenchymal stem cells attenuate blood-brain barrier leakage after cerebral ischemia in mice. J Neuroinflammation 2018;151(1).
84. Abd El Motteleb DM, Hussein S, Hasan MM and Mosaad H. Comparison between the effect of human Wharton's jelly-derived mesenchymal stem cells and levetiracetam on brain infarcts in rats. J. Cell. Biochem. 2018;11912(12).
85. Cherkashova EA, Burunova VV, Bukharova TB et al. Comparative Analysis of the Effects of Intravenous Administration of Placental Mesenchymal Stromal Cells and Neural Progenitor Cells Derived from Induced Pluripotent Cells on the Course of Acute Ischemic Stroke in Rats. Bull. Exp. Biol. Med. 2019;1664(4).
86. Park HW, Chang JW, Yang YS et al. The Effect of Donor-Dependent Administration of Human Umbilical Cord Blood-Derived Mesenchymal Stem Cells following Focal Cerebral Ischemia in Rats. Exp Neurobiol 2015;244(4).
87. Park HW, Kim Y, Chang JW et al. Effect of Single and Double Administration of Human Umbilical Cord Blood-Derived Mesenchymal Stem Cells Following Focal Cerebral Ischemia in Rats. Exp Neurobiol 2017;261(1).
88. Hou B, Ma J, Guo X et al. Exogenous Neural Stem Cells Transplantation as a Potential Therapy for Photothrombotic Ischemia Stroke in Kunming Mice Model. Mol. Neurobiol. 2017;542(2).
89. Liu D, Ye Y, Xu L, Yuan W and Zhang Q. Icariin and mesenchymal stem cells synergistically promote angiogenesis and neurogenesis after cerebral ischemia via PI3K and ERK1/2 pathways. Biomed. Pharmacother. 2018;108.
90. Chi L, Huang Y, Mao Y et al. Tail Vein Infusion of Adipose-Derived Mesenchymal Stem Cell Alleviated Inflammatory Response and Improved Blood Brain Barrier Condition by Suppressing Endoplasmic Reticulum Stress in a Middle Cerebral Artery Occlusion Rat Model. Med. Sci. Monit. 2018;24.
91. XIE P, DENG M, SUN QG et al. Therapeutic effect of transplantation of human bone marrow derived mesenchymal stem cells on neuron regeneration in a rat model of middle cerebral artery occlusion. Molecular Medicine Reports. 2019;20: 3065-3074.
92. Ryu S, Lee SH, Kim SU and Yoon BW. Human neural stem cells promote proliferation of endogenous neural stem cells and enhance angiogenesis in ischemic rat brain. Neural Regen Res 2016;112(2).
93. Hosseini SM, Samimi N, Farahmandnia M et al. The Preventive Effects of Neural Stem Cells and Mesenchymal Stem Cells Intra-ventricular Injection on Brain Stroke in Rats. N Am J Med Sci 2015;79(9).
94. Saraf J, Sarmah D, Vats K et al. Intra-arterial stem cell therapy modulates neuronal calcineurin and confers neuroprotection after ischemic stroke. Int. J. Neurosci. 2019;12910(10).
95. Oh SH, Jeong YW, Choi W et al. Multimodal Therapeutic Effects of Neural Precursor Cells Derived from Human-Induced Pluripotent Stem Cells through Episomal Plasmid-Based Reprogramming in a Rodent Model of Ischemic Stroke. Stem Cells Int 2020;2020.
96. Zeinab Vahidinia, Abolfazl Azami Tameh, Majid Nejati et al. The protective effect of bone marrow mesenchymal stem cells in a rat model of ischemic stroke via reducing the C-Jun N-terminal kinase expression. pathology research and practice 2019.
97. Wu KJ, Yu SJ, Chiang CW et al. Transplantation of human placenta-derived multipotent stem cells reduces ischemic brain injury in adult rats. Cell Transplant 2015;243(3).
98. Leu S, Lin YC, Yuen CM et al. Adipose-derived mesenchymal stem cells markedly attenuate brain infarct size and improve neurological function in rats. J Transl Med 2010;8.

**Table S4 Study characteristics accounting for heterogeneity of neurological function scores**

| neurological function scores |  | Number of animals | number of comparisons | Effect size (95% CI) | P | P |
| --- | --- | --- | --- | --- | --- | --- |
| Pooled estimate |  | 5200 | 141 | -3.37(-3.83, -2.90） |  |  |
| Type of ischemia | Temporary | 4497 | 121 | -3.59(-4.09, -3.09) | 0.13 | 0.057 |
|  | Permanent | 703 | 20 | -1.85(-3.84, 0.3) |  |  |
| recipient species | rat | 4848 | 127 | -3.55(-4.06, -3.03) | 0.22 | 0.108 |
|  | mouse | 352 | 14 | -1.92(-4.22, 0.39) |  |  |
| sex | Male | 4182 | 118 | -3.5(-4.00, -3.01) | 0.95 | 0.959 |
|  | female | 371 | 12 | -3.67(-5.90, -1.44) |  |  |
|  | Both | 363 | 3 | -2.48(-7.90, 2.95) |  |  |
|  | unclear | 262 | 8 | -3.01(-4.87, -1.16) |  |  |
| Anaesthetic | halothane | 706 | 19 | -4.64(-6.01, -3.28） | 0.23 | 0.154 |
|  | isoflurane | 1345 | 45 | -2.97(-3.83, -2.12） |  |  |
|  | ketamine | 475 | 16 | -3.86(-5.36, -2.37） |  |  |
|  | pentobarbital | 293 | 6 | -2.06(-3.96, -0.15） |  |  |
|  | chloral hydrate | 1155 | 32 | -3.66(-4.82, -2.51） |  |  |
|  | unclear | 917 | 17 | -3.34(-4.13, -2.54） |  |  |
|  | others | 309 | 6 |  |  |  |
| Donor species | mouse | 199 | 10 | -0.81(-1.48, -0.14） | 0.00001 | 0.189 |
|  | human | 1827 | 46 | -3.46(-4.33, -2.59） |  |  |
|  | rat | 2746 | 66 | -3.46(-4.13, -2.78） |  |  |
|  | porcine | 108 | 3 | -6.02(-7.92, -4.11） |  |  |
|  | Cell line | 317 | 12 | -3.98(-5.73, 2.24） |  |  |
| Stem cell | MSCs | 3020 | 81 | -3.31(-3.91, -2.71) | 0.59 | 0.76 |
|  | NSPCs | 1163 | 39 | -4.05(-5.73, -2.38) |  |  |
|  | others | 1017 | 21 | -3.51(-4.62, -2.40) |  |  |
| Cell manipulations | Gene-modification | 710 | 18 | -4.19(-6.39, -1.98） | 0.39 | 0.582 |
|  | none | 4490 | 123 | -3.39(-3.90, -2.87） |  |  |
| Dose range | <1*10^5 | 60 | 4 |  | 0.09 | 0.045 |
|  | (1-5)*10^5 | 1325 | 33 | -4.97(-6.72, -3.21) |  |  |
|  | >5*10^5 | 3464 | 92 | -3.29(-3.85, -2.72) |  |  |
|  | unclear | 351 | 12 | -2.98(-4.00, -1.96) |  |  |
| Time of admin | <1d | 1184 | 39 | -1.95(-2.49, -1.41) | 0.0003 | 0.315 |
|  | 1-7d | 2640 | 84 | -3.60(-4.22, -2.99) |  |  |
|  | >7d | 541 | 16 | -3.96(-5.42,-2.51) |  |  |
|  | unclear | 1236 | 19 | -3.74(-5.85, -1.63) |  |  |
| Method of administration | Stereotaxic | 2510 | 62 | -3.97(-4.88, -3.07) | 0.16 | 0.83 |
|  | Systemic | 2719 | 81 | -3.15(-3.75, -2.55) |  |  |
|  | intranasal | 53 | 3 | -2.90(-3.73, -2.07) |  |  |

**Table S5 Study characteristics accounting for heterogeneity of infarct volume**

| infarct volume |  | Number of animals | number of comparisons | Effect size (95% CI) | P | P |
| --- | --- | --- | --- | --- | --- | --- |
| Pooled estimate |  | 5200 | 141 | -11.37(-12.89, -9.85) |  |  |
| Type of ischemia | Temporary | 4497 | 121 | -11.99(-13.65, -10.33) | 0.001 | 0.243 |
|  | Permanent | 703 | 20 | -6.94(-9.6, -4.29) |  |  |
| species | rat | 4848 | 127 | -11.08(-12.67, -9.49) | 0.25 | 0.852 |
|  | mouse | 352 | 14 | -17.01(-27.01, -7.01) |  |  |
| sex | Male | 4204 | 118 | -10.96(-12.47, -9.46) | 0.00001 | 0.44 |
|  | female | 371 | 12 | -16.72(-27.05, -6.39) |  |  |
|  | Both | 363 | 3 | -12.77(-16.08, 9.46) |  |  |
|  | unclear | 262 | 8 | -3.31(-5.84, -0.78) |  |  |
| Anaesthetic | halothane | 706 | 19 | -11.67(-22.24, -1.10) | 0.0001 | 0.028 |
|  | isoflurane | 1345 | 45 | -17.09(-19.98, -14.2) |  |  |
|  | ketamine | 475 | 16 | -4.98(-9.05, -0.92) |  |  |
|  | pentobarbital | 293 | 6 | -14.16(-32.1, 3.78) |  |  |
|  | chloral hydrate | 1155 | 32 | -8.05(-10.31, 5.79) |  |  |
|  | unclear | 917 | 17 | -9.39(-11.35, -7.44) |  |  |
|  | others | 309 | 6 | -12.82(-22.75, -2.90) |  |  |
| Donor species | mouse | 199 | 10 | -15.94(-24.92, -6.96) | 0.36 | 0.097 |
|  | human | 1827 | 46 | -12.48(-16.4, -8.55) |  |  |
|  | rat | 2746 | 66 | -10.79(-12.60, -8.97) |  |  |
|  | porcine | 108 | 3 |  |  |  |
|  | Cell line | 317 | 12 | -8.13(-12.72, -3.54) |  |  |
| Stem cell | MSCs | 3020 | 81 | -10.81(-12.72, -8.9) | 0.47 | 0.623 |
|  | NSPCs | 1163 | 39 | -10.59(-14.12, -7.05) |  |  |
|  | others | 1017 | 21 | -13.50(-17.50, -9.50) |  |  |
| Cell manipulations | Gene-modification | 710 | 18 | -13.29(-20.09, -6.48） | 0.55 | 0.844 |
|  | none | 4490 | 123 | -11.27(-13.01, -9.53） |  |  |
| Dose range | <1*10^5 | 60 | 4 | -10.00(-12.95, -7.05) | 0.54 | 0.886 |
|  | (1-5)*10^5 | 1325 | 33 | -12.42(-14.65, -10.19) |  |  |
|  | >5*10^5 | 3464 | 92 | -10.64(-12.62, -8.66) |  |  |
|  | unclear | 351 | 12 | -13.61(-27.25, 0.02) |  |  |
| Time of admin | <1d | 1184 | 39 | -14.74(-18.27, -11.21) | 0.03 | 0.024 |
|  | 1-7d | 2640 | 84 | -10.39(-12.41, -8.38) |  |  |
|  | >7d | 541 | 16 | -8.74(-11.56, -5.92) |  |  |
|  | unclear | 1236 | 19 | -7.74(-11.43, -4.05) |  |  |
| Method of administration | Stereotaxic | 2510 | 62 | -9.85(-11.89, -7.82) | 0.00001 | 0.923 |
|  | Systemic | 2719 | 81 | -12.58(-15.36, -9.81) |  |  |
|  | intranasal | 53 | 3 | -2.17(-3.87, -0.47) |  |  |
